# IL-21 silencing inhibits the proliferation, growth, and migration of colorectal cancer cells via suppressing phosphorylation of ERK1/2 and STAT3

**DOI:** 10.1186/s40659-026-00692-z

**Published:** 2026-04-09

**Authors:** Ching Yi Ong, Wen Tao Xiong, Nor Suhada Anuar, Eshtiyag Abdalla Abdalkareem, Ming Thong Ong, Boon Yin Khoo

**Affiliations:** 1 Institute for Research in Molecular Medicine (INFORMM), Universiti Sains Malaysia, 11800 Penang, Malaysia; 2 Tropical Medicine Research Institute (TMRI), Khartoum 11111,, Sudan

**Keywords:** IL-21, Gene silencing, Colorectal cancer, Cell proliferation, Cell migration, Protein expression

## Abstract

**Background:**

This study aimed to elucidate the proliferative and migratory roles of interleukin-21 (IL-21) in HCT-116 and HT-29 colorectal cancer cells using a loss-of-function gene approach.

**Methods:**

The short hairpin ribonucleic acid (shRNA)- and short interfering ribonucleic acid (siRNA)-mediated approaches were used to achieve specific gene silencing in both cell lines. The downstream experiments, including gene silencing efficiency, were then assessed using Western blotting, microscopy, the 3-(4,5-dimethylthiazolyl-2)-2,5-diphenyltetrazolium bromide (MTT) assay, the transwell migration assay, cell cycle analysis, the clonogenic assay, and the wound healing assay.

**Results:**

Our results revealed that silencing the IL-21 gene significantly suppressed the viability and proliferation of HCT-116 and HT-29 cells. Although IL-21 gene silencing did not induce cell cycle arrest in either cell line, the reduced expression of proliferating cell nuclear antigen (PCNA) upon gene silencing aligned with the proliferation assay results. Additionally, the migration of HCT-116 and HT-29 cells was markedly attenuated upon gene silencing. The restoration of E-cadherin expression in IL-21-silenced cell lines, compared to control cells, indicated decreased receptiveness to epithelial-to-mesenchymal transition (EMT) initiation. Furthermore, IL-21 signalling was found to promote the proliferation and migration of colorectal cancer cells by suppressing the activation and phosphorylation of ERK1/2 and STAT3.

**Conclusion:**

The pro-tumourigenic properties of IL-21 could serve as a biomarker of tumour aggressiveness in colorectal cancer and provide insights for the development of advanced cancer therapies.

## Introduction

Colorectal cancer ranks as the third most commonly diagnosed cancer after breast and lung cancers [1]. According to the GLOBOCAN database, in 2020, the burden of colorectal cancer is predicted to rise to 3.2 million new cases and 1.6 million deaths by 2040 [2]. Among the more than one million new colorectal cancer cases diagnosed annually, only approximately 20% result from heritable genetic changes. In contrast, the majority have been linked to environmental factors, such as exposure to mutagens, specific intestinal pathogens, and inflammatory bowel disease (IBD), all of which contribute to tumour development [3]. Colitis-associated colorectal cancer, a high-mortality cancer subtype, is associated with IBD. Some evidence suggests that inflammation-induced tumour metastasis is a key factor contributing to the high mortality rate of colorectal cancer [4].

In the 1800s, cancer was associated with inflammation at both its initial and progressive stages [5]. In recent decades, extensive genetic, pharmacological, and epidemiological evidence has reinforced the link between inflammation and the development of colorectal cancer [3]. Inflammatory conditions can drive oncogenesis, while genetic and epigenetic changes in malignant cells can create an inflammatory microenvironment that promotes tumour formation [5]. Recently, several studies have sought to expand our understanding of the molecular mechanisms underlying inflammation-driven cancer development. In particular, inflammatory mediators, such as cytokines, which play a crucial role in cancer-associated inflammation, have garnered significant research interest, offering crucial insights into the development of targeted cancer therapies.

Interleukin-21 (IL-21) was first identified as a cytokine produced by activated CD4^+^ T cells. Cytokines induce the proliferation of both T and B cells, thereby activating natural killer cells [6, 7]. IL-21 is known as a pleiotropic cytokine owing to its ability to stimulate both innate and adaptive immunity [8]. However, its role in cancer remains ambiguous, as it has demonstrated contrasting effects on cancer pathogenesis. Consequently, the involvement of IL-21 in cancer immunotherapy requires further investigation to fully elucidate its role in carcinogenesis. IL-21 has been shown to sustain an inflammatory circuit that promotes colorectal cancer development owing to its ability to induce Th17 cell responses. The activation of Th17 cells leads to the production of various pro-inflammatory cytokines, such as IL-17A and IL-22, which exacerbate inflammation. These cytokines contribute to carcinogenesis by inducing oxidative stress, promoting epithelial cell proliferation, and stimulating angiogenesis [9]. The findings suggest that IL-21 plays a pathogenic role in chronic inflammation and is associated with an increased risk of malignancy [10, 11]. Our previous study demonstrated that IL-21 was highly expressed in the serum of patients with colorectal cancer, particularly those with schistosomiasis-related diseases [12]. The upregulation of IL-21 expression may result from host immune responses triggered during schistosomiasis infection [13, 14]. Beyond the inflammatory conditions induced by parasitic infection, it can be hypothesised that IL-21 overexpression during infection is a potential risk factor for colorectal cancer development.

IL-21 also plays diverse roles in regulating a wide range of immune cells during infection and at different stages of tumourigenesis. Most studies on IL-21 have focused on its immunomodulatory role in host defence, inflammation, and tumour immunosurveillance; however, its biological role in colorectal cancer cells remains poorly understood [15–17]. Therefore, further investigation is essential to gain a comprehensive understanding of IL-21’s role in cancer progression beyond its immunological functions in tumours.

This study aims to investigate the biological role of IL-21 in colorectal cancer progression, particularly beyond its established immunomodulatory functions. Using colorectal cancer cell lines, this study examined how IL-21 influences tumour cell behaviours, including cell growth, proliferation, migration, and invasion. In addition, the study evaluated the molecular mechanisms underlying IL-21 signalling and its potential role in inflammation-associated tumour progression. Understanding these mechanisms may provide new insights into IL-21's contribution to colorectal cancer development and identify potential targets for future therapeutic strategies.

## Materials and methods

### Culturing HCT-116 and HT-29 cells

Colorectal cancer cell lines used in the study were previously obtained from the American Type Culture Collection (ATCC). The colorectal carcinoma HCT-116 (ATCC^®^ CCL-247^™^) and colorectal adenocarcinoma HT-29 (ATCC^®^ HTB-38^™^) were adherent cell lines with epithelial morphology. The HCT-116 cell line originated from the colon of an adult male with colon cancer. In contrast, the HT-29 cell line was isolated from a primary tumour in a 44-year-old female patient with colorectal adenocarcinoma. Both HCT-116 and HT-29 cells were cultured in a complete growth medium: high-glucose Dulbecco’s Modified Eagle Medium (DMEM; Gibco, Massachusetts, USA) supplemented with 10% fetal bovine serum (FBS; Gibco, Massachusetts, USA), 100 units/mL penicillin, and 100 µg/mL streptomycin (Gibco, Massachusetts, USA) in a Class II Biosafety Cabinet (NuAire, Plymouth, USA) using proper aseptic techniques. The cells were maintained in a carbon dioxide (CO_2_) incubator (NuAire, Plymouth, USA) in a humidified atmosphere of 5% (v/v) CO_2_ at 37 °C. Cell growth was maintained by changing the growth medium every 2–3 days to replenish nutrients, and cells were subcultured when they reached 70–80% confluency.

### IL-21 gene silencing in colorectal cancer cells

- *Small interfering RNA transfection*
  The cells were seeded in 6-well culture plates (Nunc, Roskilde, Denmark) at a density of 5 × 10^5^ cells/well in antibiotic-free growth medium supplemented with FBS. The cells were incubated until reaching 70% confluence for small interfering RNA (siRNA) duplex transfection. The siRNA transfection reagent was prepared by mixing diluted IL-21 siRNA duplex solution (4 µL of IL-21 siRNA duplex [sc-39662; Santa Cruz Biotechnology, Texas, USA] + 96 µL of serum-free medium) with diluted transfection reagent solution (6 µL of Lipofectamine 2000 [Invitrogen, Massachusetts, USA] + 94 µL of serum-free medium). The mixture was incubated at room temperature for 30 min. The cells were washed with a serum- and antibiotic-free medium before transfection. For each transfection, 0.8 mL serum-free medium was added to the IL-21 siRNA duplex transfection reagent, gently mixed, and then overlaid onto the washed cells. The cells were incubated for 6 h at 37 °C in a CO_2_ incubator, after which the medium was replaced with complete growth medium. The efficiency of IL-21 gene silencing at the protein level was assessed usingWestern blotting.
- * Short hairpin RNA transduction*
  Cells were seeded in 24-well culture plates at a density of 5 × 10^4^ cells/well in antibiotic-free growth medium supplemented with FBS. The cells were incubated overnight or until they reached 50% confluence. The cells were washed with a serum- and antibiotic-free medium before transduction. A titre of 1 × 10^6^ transducing units (TU)/mL pGIPZ lentiviral short hairpin RNA (shRNA) particles carrying a puromycin-resistance gene (Dharmacon Inc., Colorado, USA) was added to the medium and evenly distributed among the cells. The lentiviral shRNA construct sequences and their respective annotations were as follows: V2LHS_81267 (shIL-21a): 5´-CTAACATGCCCTTCATGTG-3´; V2LHS_257783 (shIL-21b): 5´-CAAATACAGGAAACAATGA-3´; and V2LHS_388343 (shIL-21c): 5´-AGGGAGAAGACAGAAACAC-3´. The cells were incubated at 37 °C in a CO_2_ incubator for 6 h. After incubation, the transduction medium was replaced with complete growth medium, and the cells were further incubated for 48 h to maximise transduction efficiency. To select for successful transduced cells, puromycin selection was performed for 10–15 days by adding 1.0 µg/mL puromycin to the culture, thereby eliminating non-shRNA-transduced cells. Transduction efficiency was assessed under an Axio Observer A1/Apotome phase-contrast inverted microscope (ZEISS, Oberkochen, Germany) by observing TurboGFP (green fluorescent protein) expression. Finally, transduced cells and culture supernatants were collected to assess IL-21 gene-silencing efficiency at the protein level using Western blotting and enzyme-linked immunosorbent assay (ELISA), respectively.

### MTT cell proliferation assay

Cells were seeded in 96-well culture plates at a density of 5 × 10^3^ cells/well. Cell viability was assessed at 24, 48, and 72 h after shRNA-transduced IL-21 gene silencing. At each time point, 25 µL of 2.5 mg/mL MTT (3-[4,5-dimethylthiazol-2-yl]-2,5-diphenyltetrazolium bromide) reagent (Thermo Fisher Scientific, Massachusetts, USA) was added per well and incubated for 4 h at 37 °C. After incubation, the supernatant was aspirated, and 200 µL of dimethyl sulfoxide (DMSO) was added to each well. The plate was gently shaken to ensure the complete solubilisation of purple formazan crystals. Absorbance of the crystals in each well was measured using a Multiskan Spectrum Spectrophotometer (Thermo Fisher Scientific, Massachusetts, USA) at a primary wavelength of 570 nm and a reference wavelength of 620 nm. The percentage cell viability indicating the effect of IL-21 gene silencing on HCT-116 and HT-29 cell growth at each time point was calculated using the following formula:

$$ {\text{Percentage of cell viability}} = \frac{{{\text{Absorbance of }}\left( {{\text{silenced sample}} - {\mathrm{blank}}} \right)}}{{{\text{Absorbance of }}\left( {{\text{control sample}} - {\mathrm{blank}}} \right)}} \times 100 $$

### Cell colony formation assay

shRNA-transduced IL-21-silenced HCT-116 and HT-29 cells were seeded at a density of 2 × 10^3^ cells/well in 6-well culture plates. The complete growth medium was replaced every three days during the incubation period. After every 24 h, colonies formed on the plates were stained using the Hemacolor^®^ Rapid Staining of Blood Smear Kit (Merck Millipore, Massachusetts, USA), according to the manufacturer’s protocol. The average size and number of colonies formed by IL-21-silenced cells over 72 h were quantified using ImageJ software (Biocompare, California, USA). The percentage of colony formation, representing the relative area of IL-21-silenced cell colonies formed compared to non-IL-21-silenced cell colonies, was calculated after every 24 h of incubation. Additionally, the percentage of individual colony sizes in IL-21-silenced cells was determined relative to non-gene-silenced cells, accounting for colony density to avoid measurement bias.

### Analysis of cell cycle phases

Cells were seeded in 6-well culture plates at a density of 5 × 10^5^ cells/well and subjected to IL-21 gene silencing, as described above. Total shRNA-transduced IL-21-silenced cells were harvested after 24, 48, and 72 h of incubation. The cell suspensions were washed twice with ice-cold Phosphate-buffered saline (PBS). A total of 1 × 10^6^ cells per sample were counted and then gradually fixed in 5 mL of ice-cold 70% ethanol, added dropwise. The cells were permeabilised by incubation on ice for 1 h, then stored at -20 °C until further use. For cell cycle analysis, ethanol-fixed cell samples were centrifuged at 500 × *g* for 5 min, and the supernatant was discarded. The cell pellets were washed with ice-cold PBS to remove any residual ethanol, then resuspended in 500 µL of FxCycle^™^ PI/RNase Staining Solution (Invitrogen, Massachusetts, USA) and incubated in the dark at room temperature for 30 min. The stained samples were transferred to 5 mL sterile flow tubes. The effect of IL-21 gene silencing on cell cycle phases was analysed using a NovoCyte Quanteon Flow Cytometer (Agilent Technologies, California, USA) with gating set to 50,000 singlet cell events.

### Cell migratory functional assays

- *Wound healing assay*
  Cells were seeded in 6-well culture plates at a density of 1 × 10^6^ cells/well and subjected to IL-21 gene silencing, as described above. A linear cell-free gap was created by gently scratching the shRNA-transduced IL-21-silenced cell monolayer using a sterile 200 µL pipette tip. After scratching, the cells were rinsed with pre-warmed PBS solution to remove cell debris, and the medium was replaced with fresh complete growth medium. Gap closure was monitored, and images of marked areas were captured at 0 (control), 24, 48, and 72 h using an Optika XDS-2 inverted microscope (Optika Srl, Ponteranica, Italy). The width of the cell-free gap at each time point was measured using ImageJ software, and the percentage of gap width of IL-2-silenced cells at each time point relative to the control was calculated. The rate of gap closure represents the rate of wound healing.
- *Transwell cell migration assay*
  Cells were harvested, and 5 × 10^4^ cells were resuspended in 500 µL of 2% growth medium and subjected to IL-21 gene silencing, as described above. The shRNA-transduced IL-21-silenced cells were plated onto 24-well Falcon Transwell^®^ inserts with 8 µm pores (Corning, New York, USA). A total of 750 µL of 10% growth medium was added to the lower chamber to ensure no bubbles formed. The Transwell inserts were removed after 24, 48, and 72 h of incubation. The medium and cell debris on top of the membrane were gently removed using sterile cotton swabs. Migrated cells attached to the bottom side of the membrane, indicating chemotactic capability, were stained using the Hemacolor^®^ Rapid Staining of Blood Smear Kit, following the manufacturer’s protocol. The percentage of the migrated cell area was then quantified using ImageJ software.

### Western blotting

The harvested shRNA-transduced IL-21-silenced cells, as described above, were first collected as cell pellets on ice, and 500 μl of RIPA buffer was added. The cells were incubated on ice for 10 min. Cell lysates were then transferred into 1.5 ml microcentrifuge tubes and incubated on ice for another 30 min. The cell lysates were centrifuged for 15 min at 4 °C to pellet down the unwanted cell debris. The mixture was then centrifuged at maximum speed at 4 °C for 10 min. The clear supernatant was transferred to a fresh microcentrifuge tube for protein concentration analysis using a BCA protein assay kit or stored at − 80 °C until use. Twenty-five (25) μg of protein from each sample was analysed by sodium dodecyl sulphate–polyacrylamide gel electrophoresis (SDS-PAGE). Following electrophoresis, the proteins distributed on the gel were transferred to a nitrocellulose membrane using a semidry transfer method with a Trans-blot SD Semidry Transfer Cell (Bio-Rad Life Sciences, Hercules, USA). After protein transfer, non-specific proteins on the membrane were blocked with a 5% blocking solution for 2 h on a shaker. Then, the membrane was washed with TBST (1 × Tris-buffered saline, 0.1% Tween^®^ 20 detergent) 3 times for 10 min each. The membranes were incubated with purified mouse monoclonal antibodies for IL-21, PCNA, E-cadherin, ERK1/2, phosphorylated-ERK, STAT3, and phosphorylated-STAT3 (1:5000 dilution in TBST) at 4 °C overnight. The next morning, the membrane was rewashed with TBST, as above. After washing, the membrane was incubated with horseradish peroxidase-conjugated goat anti-mouse IgG (1:10,000 dilution in TBST) for 1 h on a shaker. The membrane was then rewashed with TBST, as above. The protein signal development process was carried out in the darkroom, whereby the membrane was first overlaid with SuperSignal^™^ West Pico chemiluminescence substrate (Thermo Fisher Scientific, Massachusetts, USA). Cling wrap was then overlaid on the wet membrane, followed by exposure to CL-XPosure^™^ X-ray film (Thermo Fisher Scientific, Massachusetts, USA) for approximately 5 min. The film was then immersed in the developer solution for a few seconds, rinsed with tap water, and fixed in the fixer solution for a few seconds. The developed film was then air-dried and photographed with the signals on the X-ray film. The intensity of protein bands was quantified using ImageJ software.

### ELISA

The levels of IL-21 in culture supernatants of shRNA-transduced IL-21-silenced cells were measured using a Human IL-21 Platinum ELISA Kit (eBioscience, Vienna, Austria), according to the manufacturer’s instructions. Each sample, standard, and blank was assayed in triplicate. Microwell strips were washed twice with 400 μL of washing buffer per well, with the buffer remaining in the wells for 10–15 s before aspiration. After the final wash, the plate was tapped on an absorbent pad to remove excess buffer. Subsequently, 100 μL of each standard dilution was added to the designated wells. For the blank wells, 100 μL of assay buffer was added. For the sample wells, 50 μL of assay buffer was followed by 50 μL of sample (10 ng/mL). Next, 50 μL of biotin-conjugate was added to all wells. The plate was covered with adhesive film and incubated at room temperature for 3 h with shaking at 4 × g. The wells were then washed four times with 400 μL washing buffer, after which 100 μL of diluted streptavidin-HRP was added to each well. The plate was incubated for 1 h at room temperature with shaking at 4 × g. After another four washes, 100 μL of TMB substrate solution was added to each well and incubated for approximately 10 min at room temperature in the dark. The reaction was terminated by adding 100 μL of stop solution to each well. Absorbance was measured at 450 nm with a reference wavelength of 620 nm using a microplate reader (TECAN Sunrise, Männedorf, Switzerland). The concentration of IL-21 in each sample was determined from the standard curve.

### Data analysis

All data were expressed as triplicate standard deviation (SD) in at least two (2) independent experiments. Statistically significant differences in mean were determined with a one-way ANOVA analysis of variance using a post-hoc Tukey test. Significant differences were considered at a p-value of less than 0.05.

## Results

### Efficiency of siRNA- and shRNA-mediated IL-21 gene silencing in HCT-116 and HT-29 cells

**Fig. 1:**
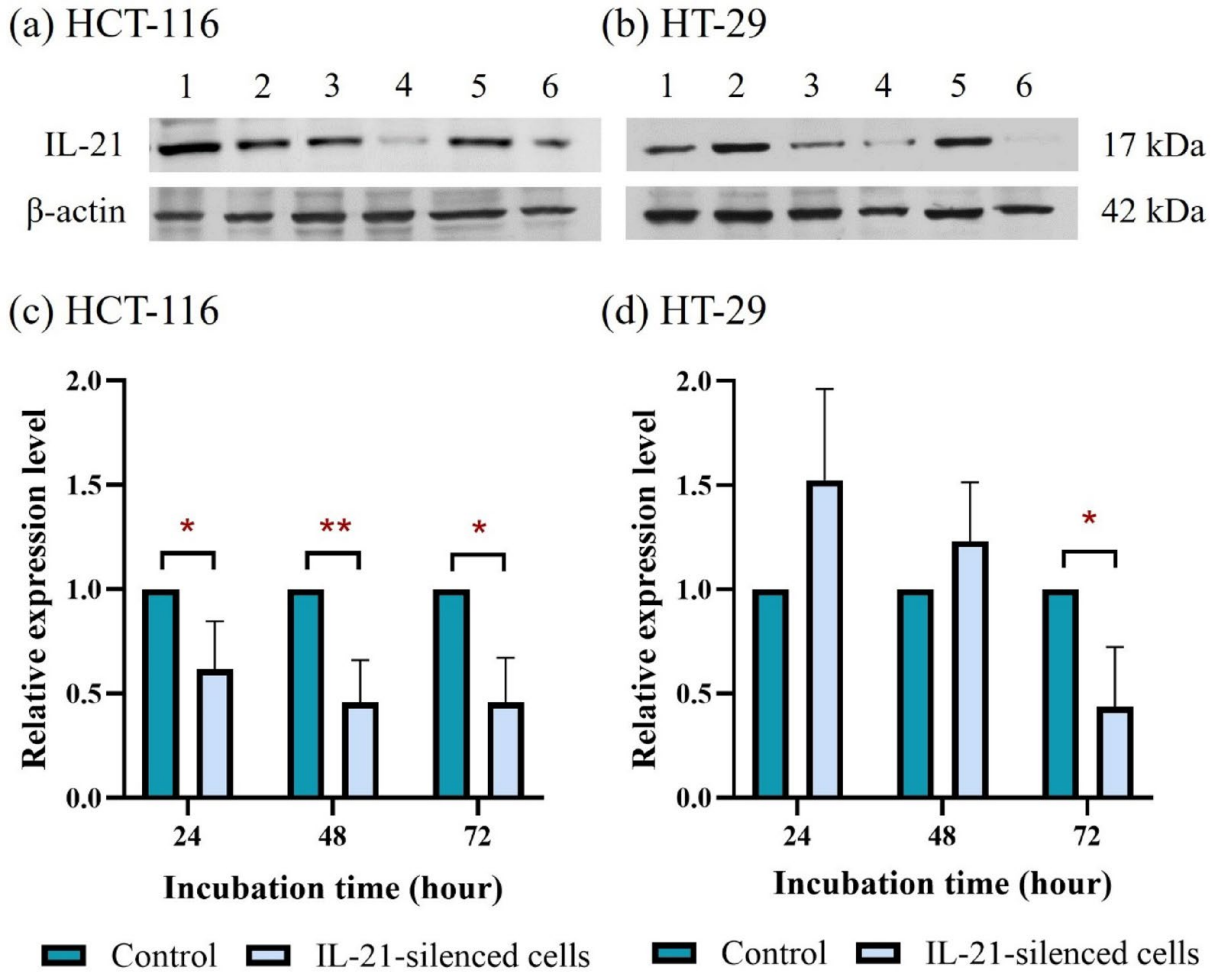
Protein expression analysis of interleukin-21 (IL-21) in HCT-116 and HT-29 cells following siRNA-mediated gene silencing. Western blot analysis of IL-21 protein bands in (**a**) HCT-116 and (**b**) HT-29 cells following siRNA-mediated IL-21 gene silencing for 24, 48 and 72 h. Lane 1: control (24 h); Lane 2: IL-21-silenced (24 h); Lane 3: control (48 h); Lane 4: IL-21-silenced (48 h); Lane 5: control (72 h); Lane 6: IL-21-silenced (72 h). Densitometric analysis of IL-21 protein expression in (**c**) HCT-116 and (**d**) HT-29 cells was performed using ImageJ software. Data are presented as mean ± standard deviation (SD) from three independent experiments and normalised to β-actin as a loading control. *P < 0.05, **P < 0.01

The efficiency of siRNA-mediated IL-21 gene silencing in HCT-116 and HT-29 cells was evaluated by measuring IL-21 protein expression over a 72h incubation period (Fig. 1). In HCT-116 cells, IL-21 protein expression was significantly downregulated at all three incubation time points. IL-21 expression decreased to 0.62-fold after 24 h (*P* < 0.05) and further declined to 0.46-fold at both 48 h (*P* < 0.01) and 72 h (*P* < 0.05) compared to non-IL-21-silenced HCT-116 cells. In contrast, siRNA-mediated IL-21 gene silencing was less effective in HT-29 cells, where significant downregulation of IL-21 protein expression was only observed after 72 h of incubation (0.44-fold; *P* < 0.05) compared to non-IL-21-silenced HT-29 cells. Overall, siRNA-mediated IL-21 gene silencing was more effective in HCT-116 cells than in HT-29 cells.

**Fig. 2:**
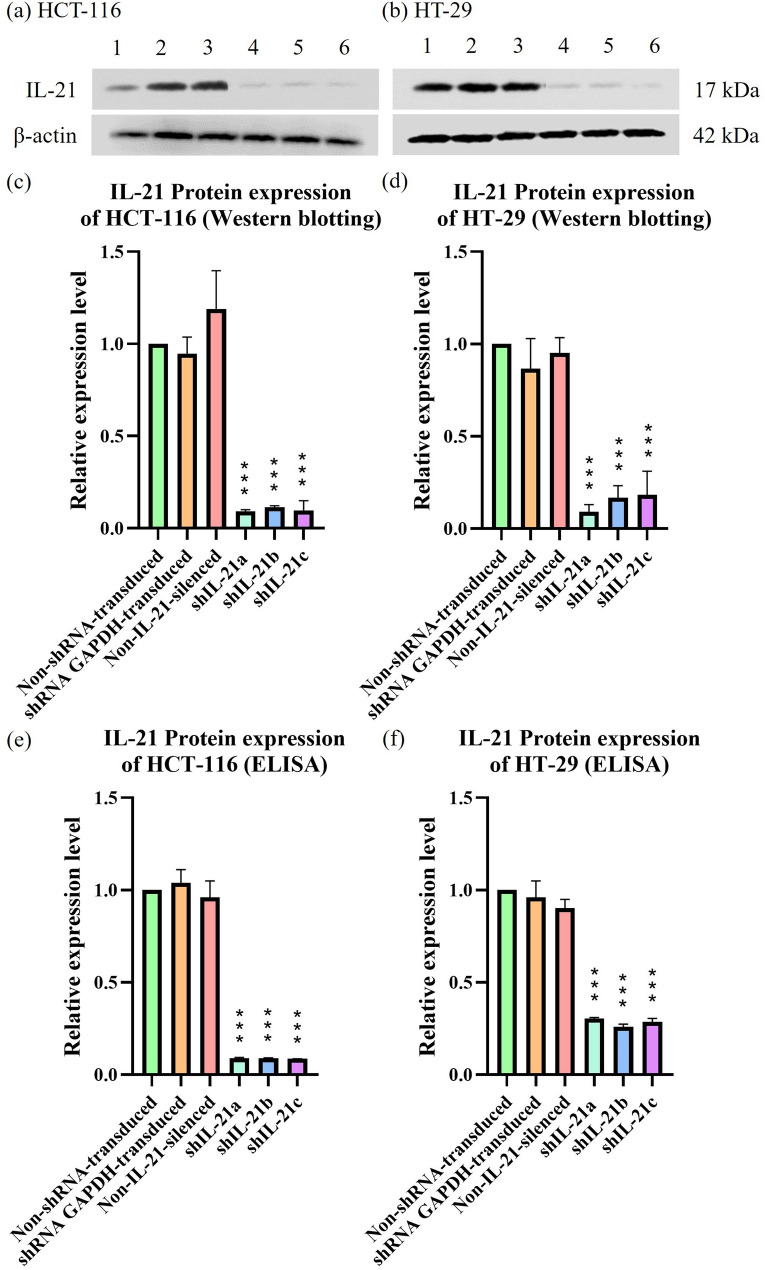
Protein expression analysis of IL-21 in HCT-116 and HT-29 cells following shRNA-transduced gene silencing. Western blot analysis of IL-21 protein bands in (**a**) HCT-116 and (**b**) HT-29 cells post shRNA-transduced gene silencing. Lane 1: non-shRNA-transduced; Lane 2: shRNA glyceraldehyde-3-phosphate dehydrogenase (GAPDH)-transduced; Lane 3: non-IL-21-silenced; Lane 4: shIL-21a; Lane 5: shIL-21b; Lane 6: shIL-21c cells. Densitometric analysis of IL-21 protein expression in (**c**) HCT-116 and (**d**) HT-29 cells was performed using ImageJ software. ELISA analysis of IL-21 protein bands in (**e**) HCT-116 and (**f**) HT-29 cell culture supernatants post shRNA-transduction for gene silencing. Data are presented as mean ± SD of three independent experiments and normalised to β-actin as a loading control. ***P < 0.001

shRNA-mediated IL-21 gene silencing resulted in a marked downregulation of IL-21 protein expression in HCT-116 cells compared with non-shRNA-transduced cells (Fig. 2). After 48 h of incubation, IL-21 protein expression was reduced to 0.09-fold (P < 0.001; shIL-21a), 0.11-fold (P < 0.001; shIL-21b), and 0.10-fold (P < 0.001; shIL-21c). No significant change in IL-21 expression was observed in shRNA glyceraldehyde-3-phosphate dehydrogenase (GAPDH)-transduced or non-IL-21-silenced HCT-116 cells. Similarly, in HT-29 cells, shRNA-mediated IL-21 gene silencing significantly reduced IL-21 protein expression to 0.09-fold (P < 0.001; shIL-21a), 0.17-fold (P < 0.001; shIL-21b), and 0.18-fold (P < 0.001; shIL-21c) after 48 h of incubation, while IL-21 expression remained unchanged in shRNA GAPDH-transduced and non-IL-21-silenced HT-29 cells. A comparable reduction was observed in the culture supernatants. In HCT-116 cell supernatants, IL-21 levels decreased to 0.089-fold (P < 0.001; shIL-21a), 0.088-fold (P < 0.001; shIL-21b), and 0.085-fold (P < 0.001; shIL-21c) after 48 h of incubation, with no significant changes detected in shRNA GAPDH-transduced or non-IL-21-silenced HCT-116 cells. In HT-29 cell supernatants, IL-21 protein expression was similarly reduced to 0.303-fold (P < 0.001; shIL-21a), 0.260-fold (P < 0.001; shIL-21b), and 0.286-fold (P < 0.001; shIL-21c), while shRNA GAPDH-transduced and non-IL-21-silenced HT-29 cells showed no significant changes. Overall, these results confirm that shRNA-mediated IL-21 gene silencing was highly effective in both HCT-116 and HT-29 cells, resulting in strong and sustained knockdown under puromycin selection.

### shRNA-transduced IL-21 gene silencing in HCT-116 and HT-29 cells

**Fig. 3:**
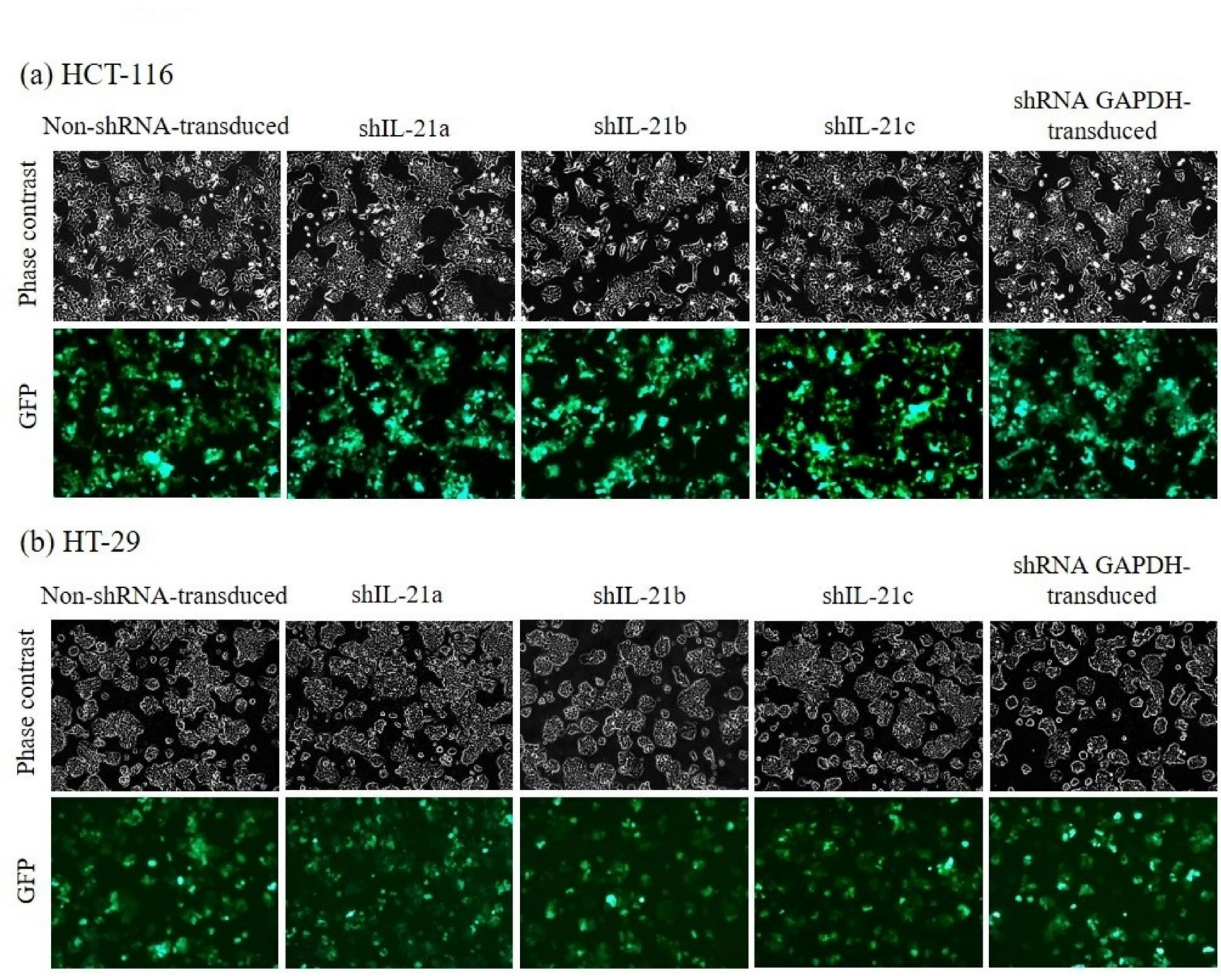
shRNA-transduced IL-21 gene silencing in HCT-116 and HT-29 cells. Phase-contrast and fluorescent images of (**a**) HCT-116 and (**b**) HT-29 cells were captured using the Axio Observer A1/Apotome phase-contrast inverted microscope at 100 × magnification. GFP-positive cells in non-shRNA-transduced, shRNA IL-21-transduced (shIL-21a, shIL-21b, shIL-21c) and shRNA GAPDH-transduced cells.

The percentage of GFP-positive cells, indicating the transduction efficiency of the shRNA lentiviral vector in HCT-116 and HT-29 cells at 24, 48, and 72 h, is shown in Fig. 3. The three IL-21 shRNAs (shIL-21a, shIL-21b, and shIL-21c), along with positive and negative controls, achieved transduction efficiencies of at least 80% in HCT-116 cells and 75% in HT-29 cells. The positive control consisted of cells transduced with shRNA GAPDH, while the negative control consisted of non-shRNA-transduced cells. Western blot and ELISA analyses confirmed the efficiency of shRNA-mediated IL-21 genesilencing in both cell lines.

### Cell viability of shRNA-transduced IL-21 gene silencing in HCT-116 and HT-29 cells

**Fig. 4:**
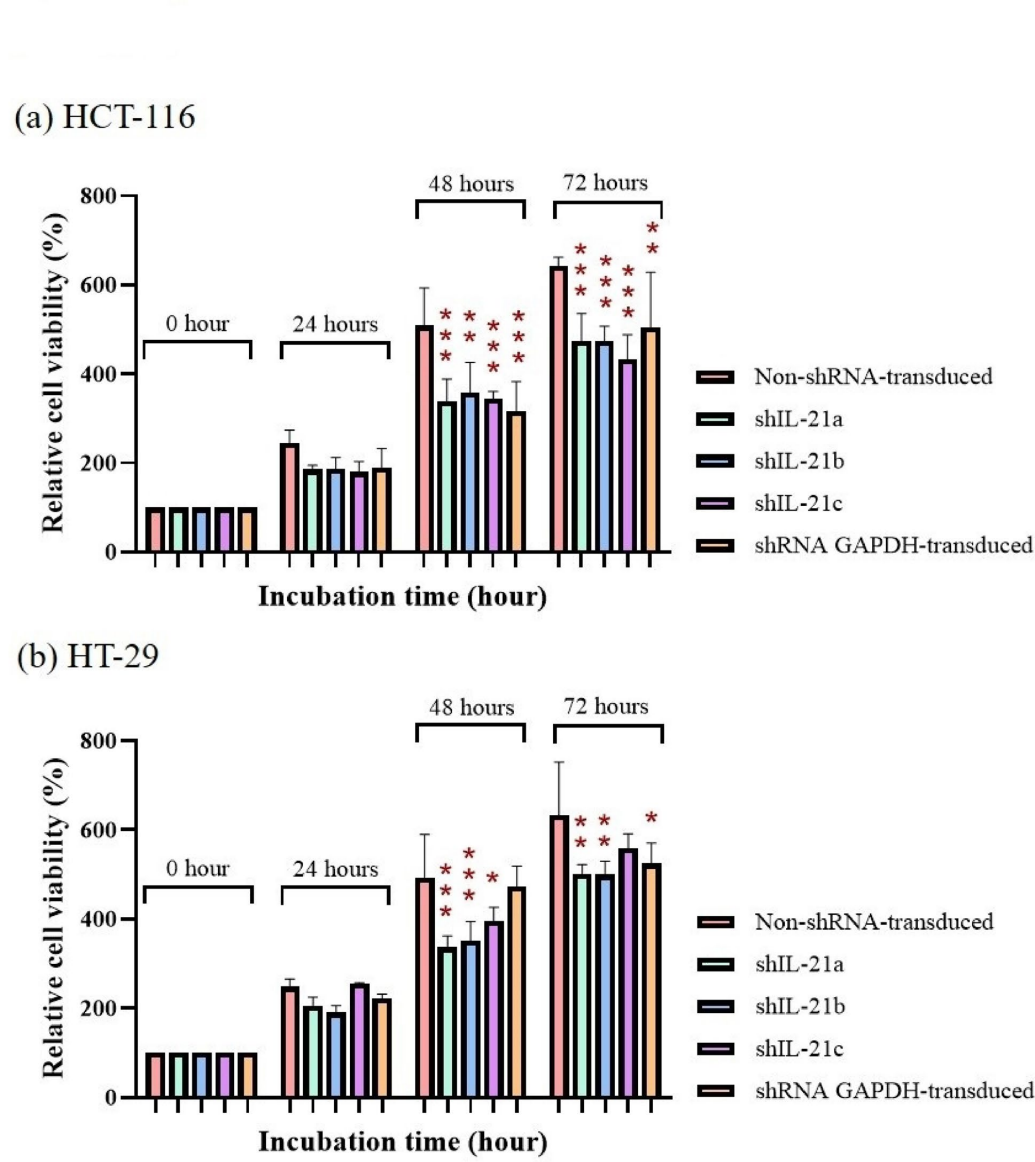
Cell viability of IL-21-silenced HCT-116 and HT-29 cells. The effect of shRNA-transduced IL-21 gene silencing on cell viability in (**a**) HCT-116 and (**b**) HT-29 cells was assessed at 24, 48 and 72 h of incubation. Absorbance values at each time point were normalised to the initial time point (0 h). Data are presented as mean ± SD of three independent experiments. *P < 0.05, **P < 0.01, ***P < 0.001

The effects of shRNA-mediated IL-21 gene silencing on the viability of HCT-116 and HT-29 cells after 72 h of incubation are presented in Fig. 4. IL-21-silenced HCT-116 cells exhibited significantly reduced cell viability after 24 h, with the difference becoming more pronounced after 48 and 72 h compared to non-shRNA-transduced cells (control). After 48 h, control cells reached 508.70% cell viability relative to the initial time point, whereas IL-21-silenced HCT-116 cells showed significantly lower viabilities of 339.15% (*P* < 0.001; shIL-21a), 357.53% (*P* < 0.01; shIL-21b), and 344.63% (*P* < 0.001; shIL-21c). By 72 h, control cells attained 643.65% viability, while IL-21-silenced HCT-116 cells achieved only 475.28% (*P* < 0.001; shIL-21a), 474.57% (*P* < 0.001; shIL-21b), and 432.39% (*P* < 0.001; shIL-21c), confirming that shRNA-mediated IL-21 gene silencing decreased the viability of HCT-116 cells. A similar result was observed in shRNA GAPDH-transduced HCT-116 cells, with significantly reduced viability at 48 h (317.22%; *P* < 0.001) and 72 h (504.34%; *P* < 0.01).

Likewise, IL-21-silenced HT-29 cells showed reduced cell viability compared to control cells at all three time points. The reduction in viability of IL-21-silenced HT-29 cells at 24 h was not statistically significant. However, the difference in viability between control and IL-21-silenced HT-29 cells increased and was statistically significant at 48 and 72 h. At 48 h, control cells reached 493.34% viability, whereas IL-21-silenced HT-29 cells showed lower viabilities of 337.21% (*P* < 0.001; shIL-21a), 352.61% (*P* < 0.001; shIL-21b), and 395.42% (*P* < 0.05; shIL-21c). In contrast, by 72 h, control cells reached 632.73% viability, whereas IL-21-silenced HT-29 cells achieved a cell viability of only 501.84% (*P* < 0.01; shIL-21a), 499.37% (*P* < 0.01; shIL-21b), and 558.19% (   shIL-21c). A minor reduction in viability was also observed in shRNA GAPDH-transduced HT-29 cells after 48 h (473.79%) and 72 h (524.86%; *P* < 0.05). Overall, these findings indicate that shRNA-transduced IL-21 gene silencing reduced cell viability in both HCT-116 and HT-29 cells at 48 h and 72 h compared to control cells.

### Clonogenicity of shRNA-transduced IL-21 gene silencing in HCT-116 and HT-29 cells

**Fig. 5:**
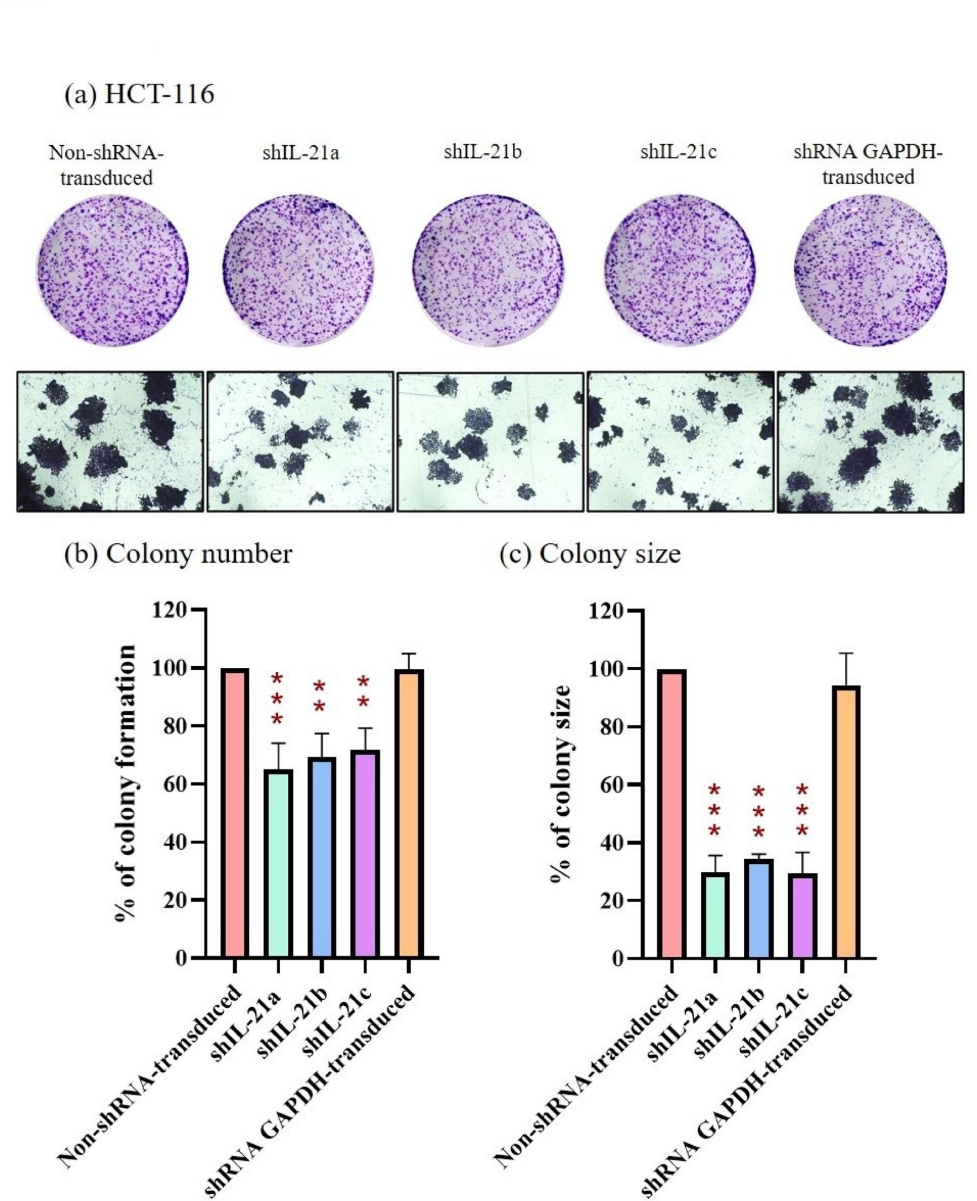
Clonogenicity of shRNA-transduced IL-21-silenced HCT-116 cells. (**a**) HCT-116 colonies stained using the Hemacolor^®^ Rapid Staining of Blood Smear Kit after 1 week of incubation. Images were captured using an Optika XDS-2 inverted microscope at 1 × and 40 × magnifications. (**b**) Colony formation and (**c**) colony size percentages of IL-21-silenced HCT-116 cells were quantified using ImageJ software. Data are presented as mean ± SD of three independent experiments. **P < 0.01, ***P < 0.001

The colony formation assay assessed the effect of shRNA-transduced IL-21 gene silencing on the clonogenicity of both HCT-116 and HT-29 cells. A reduction in colony formation was observed in IL-21-silenced HCT-116 cells compared to that in non-shRNA-transduced cells (control) after a week of incubation (Fig. 5a). Specifically, IL-21-silenced HCT-116 cells exhibited colony formation percentages of only 65.19% (*P* < 0.001; shIL-21a), 69.31% (*P* < 0.01; shIL-21b), and 71.87% (*P* < 0.01; shIL-21c) relative to the control (100%), whereas shRNA GAPDH-transduced HCT-116 cells maintained 99.59% colony formation (Fig. 5b). Additionally, IL-21-silenced HCT-116 cells displayed a substantial reduction in colony size, with decreases of 70.13% (*P* < 0.001; shIL-21a), 65.56% (*P* < 0.001; shIL-21b), and 70.51% (*P* < 0.001; shIL-21c), whereas shRNA GAPDH-transduced HCT-116 cells showed only a 5.53% reduction (94.47%) compared to the control (100%; Fig. 5c). These findings indicate that shRNA-transduced IL-21 gene silencing impaired the clonogenicity of HCT-116 cells.

**Fig. 6:**
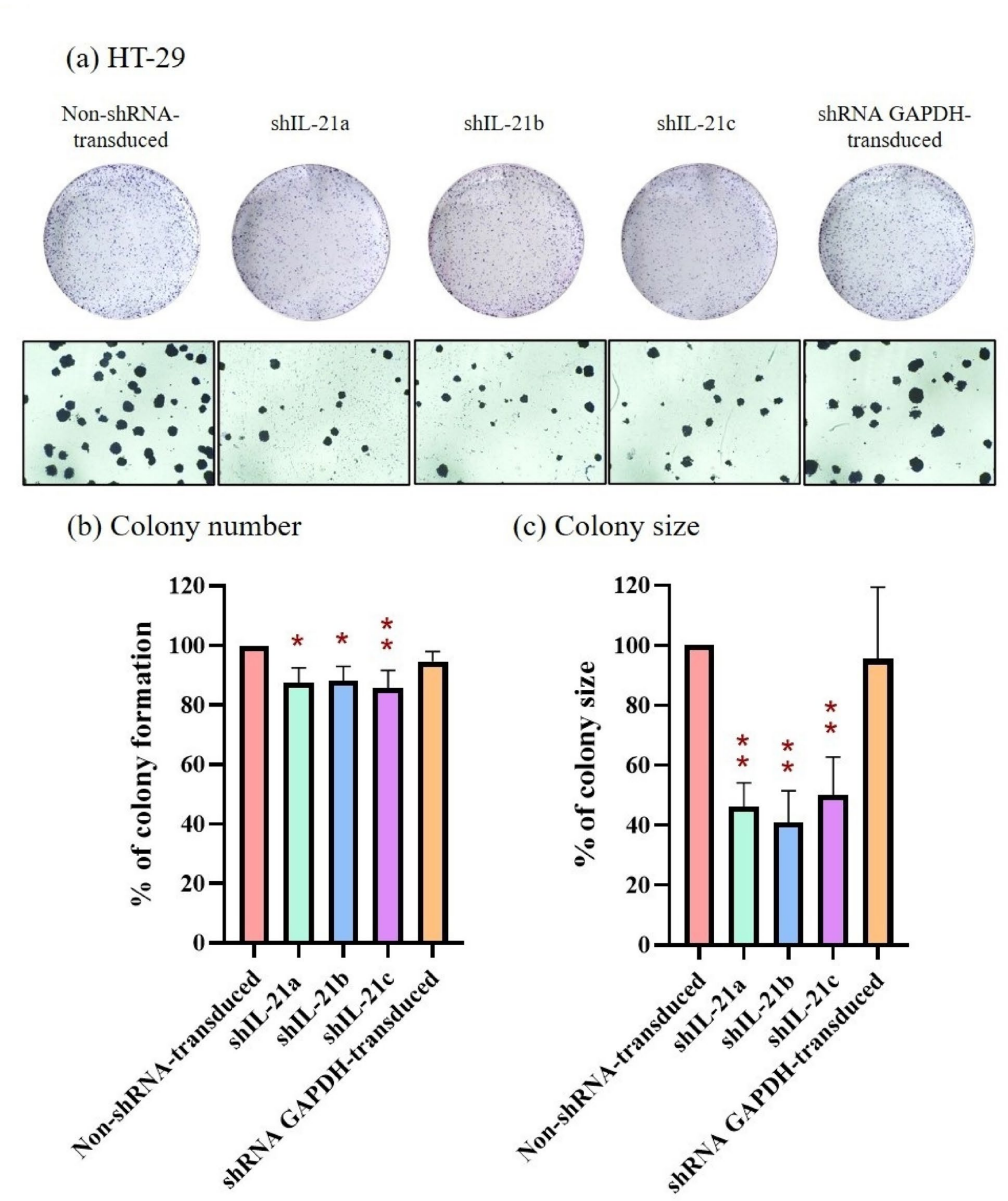
Clonogenicity of shRNA-transduced IL-21-silenced HT-29 cells. (**a**) HT-29 colonies stained using the Hemacolor^®^ Rapid Staining of Blood Smear Kit after 1 week of incubation. Images were captured using an Optika XDS-2 inverted microscope at 1 × and 40 × magnifications. (**b**) Colony formation and (**c**) colony size percentages of IL-21-silenced HT-29 cells were quantified using ImageJ software. Data are presented as mean ± SD of three independent experiments. *P < 0.05, **P < 0.01

Similarly, Fig. 6a demonstrates reduced colony formation in IL-21-silenced HT-29 cells after a week of incubation. IL-21-silenced HT-29 cells formed fewer colonies, achieving 87.56% (*P* < 0.05; shIL-21a), 88.22% (*P* < 0.05; shIL-21b), and 85.77% (*P* < 0.01; shIL-21c) colony formation compared to non-shRNA-transduced cells (control; 100%), whereas shRNA GAPDH-transduced HT-29 cells maintained 94.73% colony formation (Fig. 6b). Although the reduction in colony formation of HT-29 cells was less pronounced than that in HCT-116 cells, the effect remained statistically significant. Similar to HCT-116 cells, the average colony size of HT-29 cells was significantly affected by IL-21 gene silencing, with a decrease of 53.90% (*P* < 0.01; shIL-21a), 59.08% (*P* < 0.01; shIL-21b), and 49.88% (*P* < 0.01; shIL-21c) in the sizes of colony, whereas shRNA GAPDH-transduced HT-29 cells showed only a 4.52% reduction compared to the control (100%; Fig. 6c). Overall, these results demonstrate that shRNA-transduced IL-21 gene silencing reduces clonogenicity in both HCT-116 and HT-29 cells, with a more pronounced effect observed in HCT-116 cells than in HT-29 cells.

### Cell cycle phase distribution of shRNA-transduced IL-21-silenced HCT-116 and HT-29 cells

**Fig. 7:**
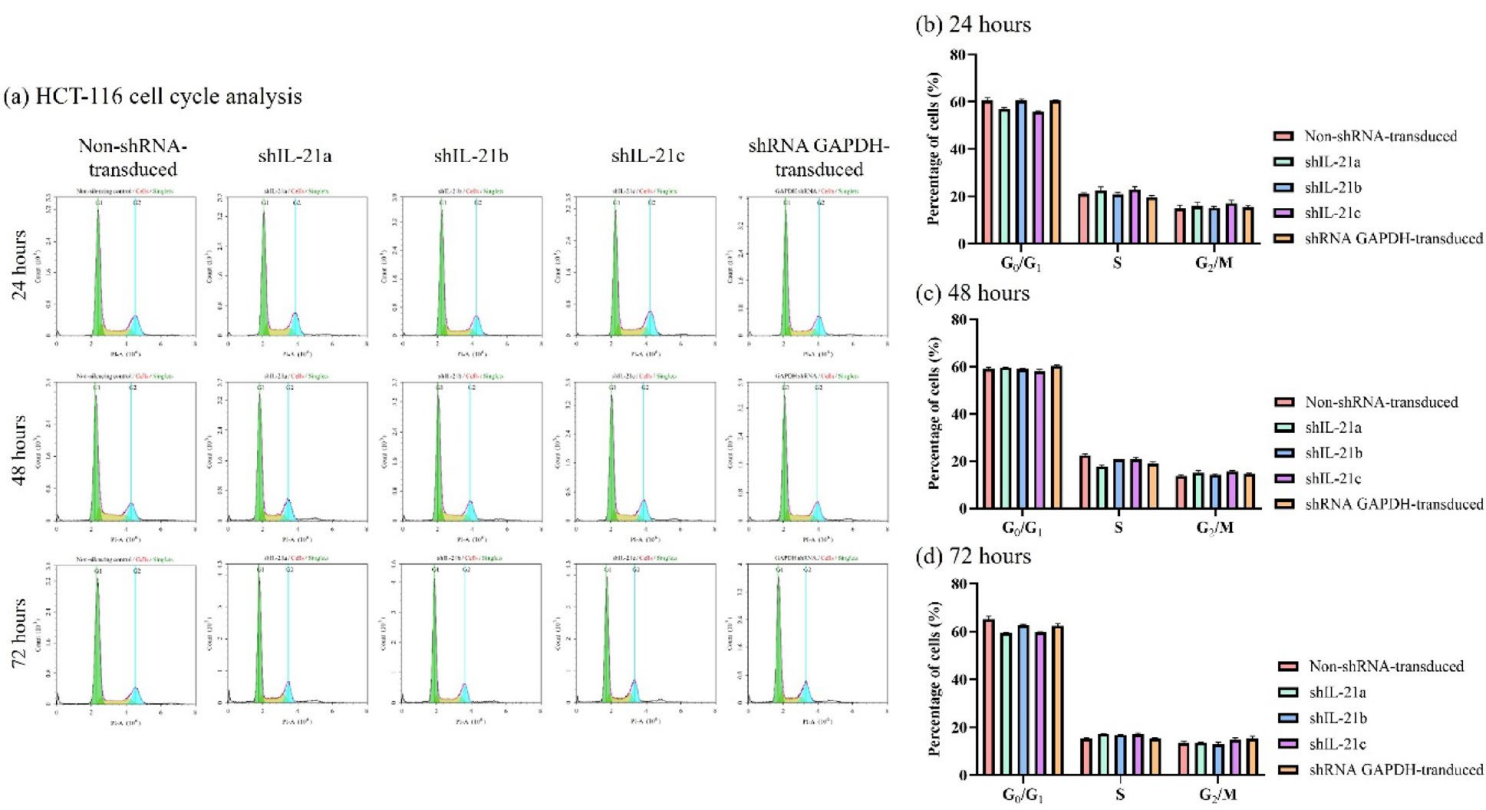
Cell cycle phase distribution of shRNA-transduced IL-21-silenced HCT-116 cells. (**a**) Cell cycle plots of non-shRNA-transduced and IL-21-silenced HCT-116 cells at 24, 48 and 72 h of incubation. The plots were generated using NovoExpress flow cytometry software version 1.5.6. Bar charts represent the percentage of HCT-116 cells in the G0/G1, S, and G2/M phases at (**b**) 24, (**c**) 48 and (**d**) 72 h. Data are presented as mean ± SD of three independent experiments

**Fig. 8:**
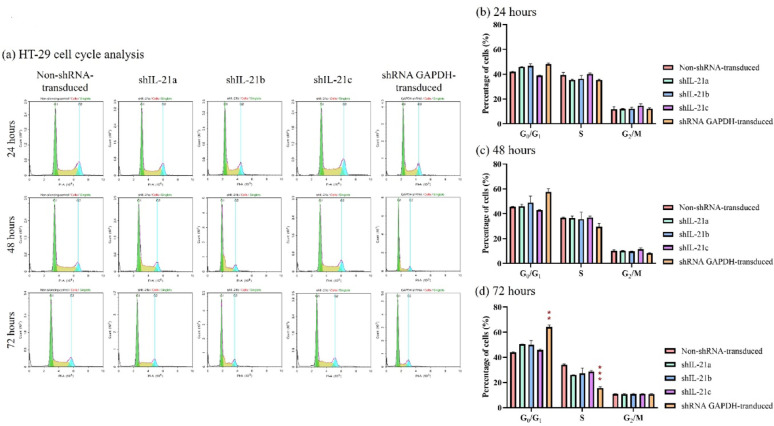
Cell cycle phase distribution of shRNA-transduced IL-21-silenced HT-29 cells. (**a**) Cell cycle plots of non-shRNA-transduced and IL-21-silenced HT-29 cells at 24, 48 and 72 h of incubation. The plots were generated using NovoExpress flow cytometry software version 1.5.6. Bar charts represent the percentage of HT-29 cells in the G0/G1, S, and G2/M phases at (**b**) 24, (**c**) 48 and (**d**) 72 h. Data are presented as mean ± SD of three independent experiments. *P < 0.05, **P < 0.01

Cell cycle analysis revealed that shRNA-transduced IL-21gene silencing did not significantly alter the cell cycle phase distribution in HCT-116 cells at any of the three time points compared to non-shRNA transduced cells (Fig. 7). This suggests that IL-21 gene silencing did not induce cell cycle arrest in HCT-116 cells. In addition, shRNA GAPDH-transduced HCT-116 cells also showed no significant changes in cell cycle phase distribution. Similarly, there was no corresponding accumulation or reduction in HT-29 cells across any cell cycle phase after 24 h and 48 h. However, after 72 h, shRNA GAPDH-transduced HT-29 cells exhibited a significant increase (64.05%; *P* < 0.01) in the G0/G1 phase (Fig. 8), accompanied by a significant reduction (15.93%; *P* < 0.001) in the S phase population compared to the non-shRNA transduced HT-29 cells. This accumulation in the G0/G1 phase suggests that shRNA-GAPDH-transduced HT-29 cells experienced cell-cycle arrest at this stage. Overall, IL-21 gene silencing did not induce cell cycle arrest in either HCT-116 or HT-29 cells. Therefore, the previously observed reductions in cell viability and proliferation rates in shRNA-transduced IL-21-silenced cells were not attributable to cell cycle arrest.

### Wound healing ability of shRNA-transduced IL-21-silenced HCT-116 and HT-29 cells

**Fig. 9:**
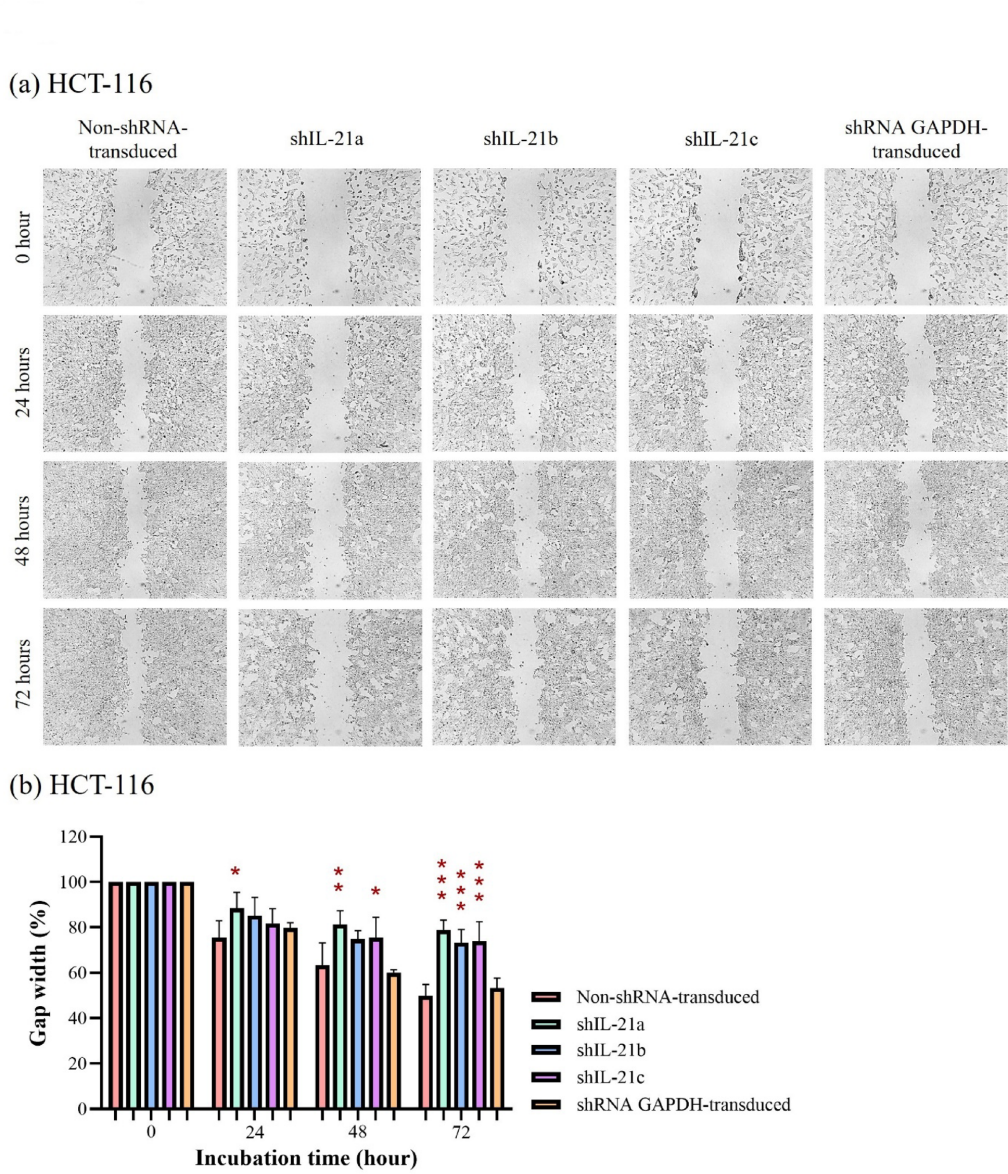
Wound healing assay of shRNA-transduced IL-21-silenced HCT-116 cells. (**a**) Representative images of wound gaps in IL-21-silenced HCT-116 cells captured using an Optika XDS-2 inverted microscope at 40 × magnification. The average wound gap widths in IL-21-silenced HCT-116 cells were quantified at 24, 48 and 72 h of incubation using ImageJ software. (**b**) The percentage of gap width at each time point was calculated relative to the initial gap width (0 h). Data are presented as mean ± SD of three independent experiments. *P < 0.05, **P < 0.01, ***P < 0.001

The wound healing assay revealed that shRNA-transduced IL-21-silenced HCT-116 cells exhibited a slower wound closure rate than non-shRNA transduced HCT-116 cells (control) at all time points. After 24 h, IL-21-silenced HCT-116 cells retained 88.45% (*P* < 0.05; shIL-21a) of the initial gap width, whereas the control and shRNA GAPDH-transduced HCT-116 cells displayed gap widths of only 75.54% and 79.76%, respectively (Fig. 9a, b). At 48 h, the gap width in IL-21-silenced HCT-116 cells decreased to 81.30% (*P* < 0.01; shIL-21a) and 75.58% (*P* < 0.05; shIL-21c), whereas the control and shRNA GAPDH-transducedHCT-116 cells showed further closure, reaching 63.36% and 60.00% of the original gap width, respectively. After 72 h, the gap widths of IL-21-silenced HCT-116 cells remained at 78.90% (*P* < 0.001; shIL-21a), 73.30% (*P* < 0.001; shIL-21b), and 73.86% (*P* < 0.001; shIL-21c), whereas the control and shRNA GAPDH-transduced HCT-116 cells dropped to 49.89% and 53.38%, respectively.

**Fig. 10:**
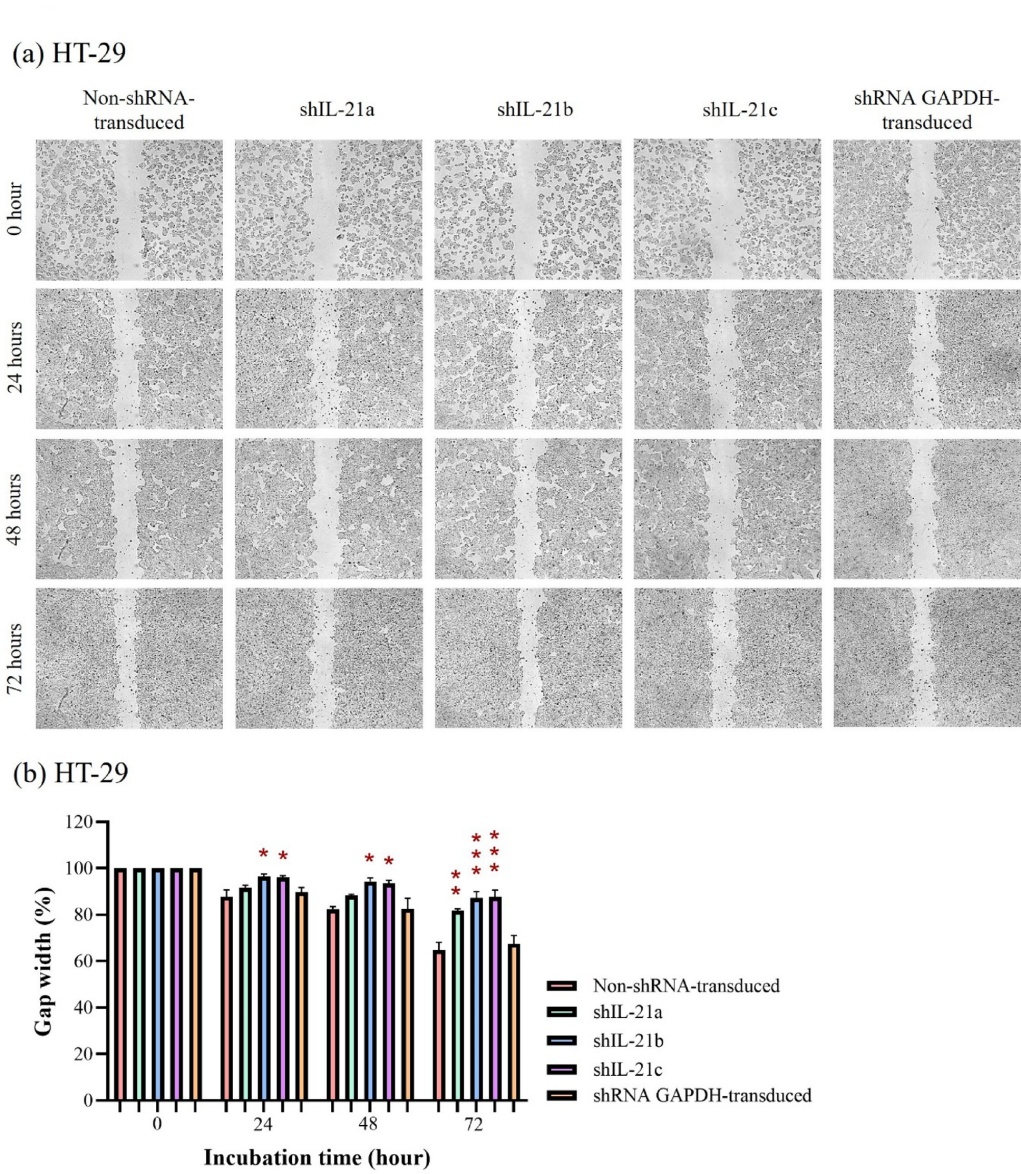
Wound healing assay of shRNA-transduced IL-21-silenced HT-29 cells. (**a**) Representative images of wound gaps in IL-21-silenced HT-29 cells captured using an Optika XDS-2 inverted microscope at 40 × magnification. The average wound gap widths in IL-21-silenced HT-29 cells were quantified at 24, 48 and 72 h of incubation using ImageJ software. (**b**) The percentage of gap width at each time point was calculated relative to the initial gap width (0 h). Data are presented as mean ± SD of three independent experiments. *P < 0.05, **P < 0.01, ***P < 0.001

In contrast, IL-21-silenced HT-29 cells retained 96.59% (*P* < 0.05; shIL-21b) and 96.14% (*P* < 0.05; shIL-21c) of the initial gap width, whereas non-shRNA transduced (control) and shRNA GAPDH-transduced HT-29 cells displayed gap widths of 87.85% and 89.73%, respectively, at 24 h (Fig. 10a, b). After 48 h, the gap width in IL-21-silenced HT-29 cells decreased to 94.38% (*P* < 0.05; shIL-21b) and 93.44% (*P* < 0.05; shIL-21c), whereas that of the control and shRNA GAPDH-transduced HT-29 cells was 82.50% and 82.53%, respectively. By 72 h, IL-21-silenced HT-29 cells reached 81.71% (*P* < 0.01; shIL-21a), 87.23% (*P* < 0.001; shIL-21b), and 87.74% (*P* < 0.001; shIL-21c) of the initial width, while the control and shRNA GAPDH-transduced HT-29 cells displayed significant wound closure, reaching 64.81% and 67.60%, respectively. Overall, shRNA-transduced IL-21-silenced HCT-116 cells exhibited significantly reduced wound healing ability compared to HT-29 cells. However, both cell lines followed a similar trend, showing a statistically significant reduction in migratory activity upon IL-21 gene silencing.

### Chemotactic capability of shRNA-transduced IL-21-silenced HCT-116 and HT-29 cells

**Fig. 11:**
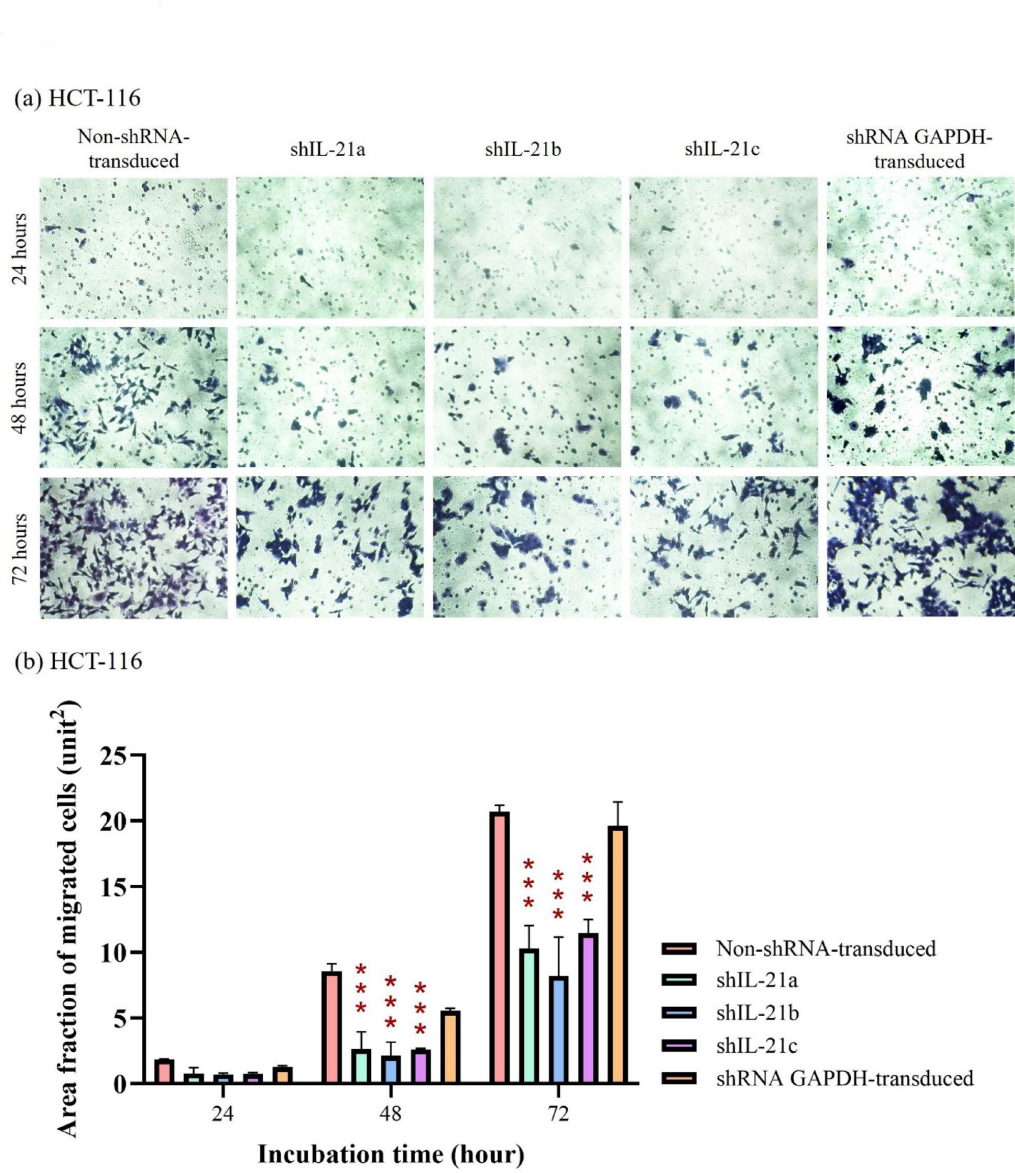
Migration analysis of shRNA-transduced IL-21-silenced HCT-116 cells. (**a**) Representative images of IL-21-silenced HCT-116 cells that migrated through an 8.0 µm pore size polyester membrane, captured using an Optika XDS-2 inverted microscope at 200 × magnification. (**b**) Area fractions of migrated IL-21-silenced HCT-116 cells stained using the Hemacolor^®^ Rapid Staining of Blood Smear Kit at 24, 48 and 72 h of incubation, quantified using ImageJ software. Data are presented as mean ± SD of three independent experiments. *P < 0.05, **P < 0.01, ***P < 0.0001

The Transwell migration assay demonstrated that the area fraction of shRNA-transduced IL-21-silenced HCT-116 cells migrating through the porous membrane was lower than that of non-shRNA-transduced (control) and shRNA GAPDH-transduced HCT-116 cells at all time points (Fig. 11a, b). After 48 h, the area fraction of migrated IL-21-silenced HCT-116 cells was significantly reduced to 2.63 (*P* < 0.001; shIL-21a), 2.13 (*P* < 0.001; shIL-21b), and 2.59 unit^2^ (*P* < 0.001; shIL-21c), whereas the control and shRNA GAPDH-transduced HCT-116 cells showed higher migration rates of 8.58 and 5.55 unit^2^, respectively. By 72 h, the area fraction of migrated cells remained lower at 10.28 (*P* < 0.001; shIL-21a), 8.19 (*P* < 0.001; shIL-21b) and 11.47 unit^2^ (*P* < 0.001; shIL-21c) compared to that of the control (20.70 unit^2^) and shRNA GAPDH-transduced HCT-116 cells (19.63 unit^2^).

**Fig. 12:**
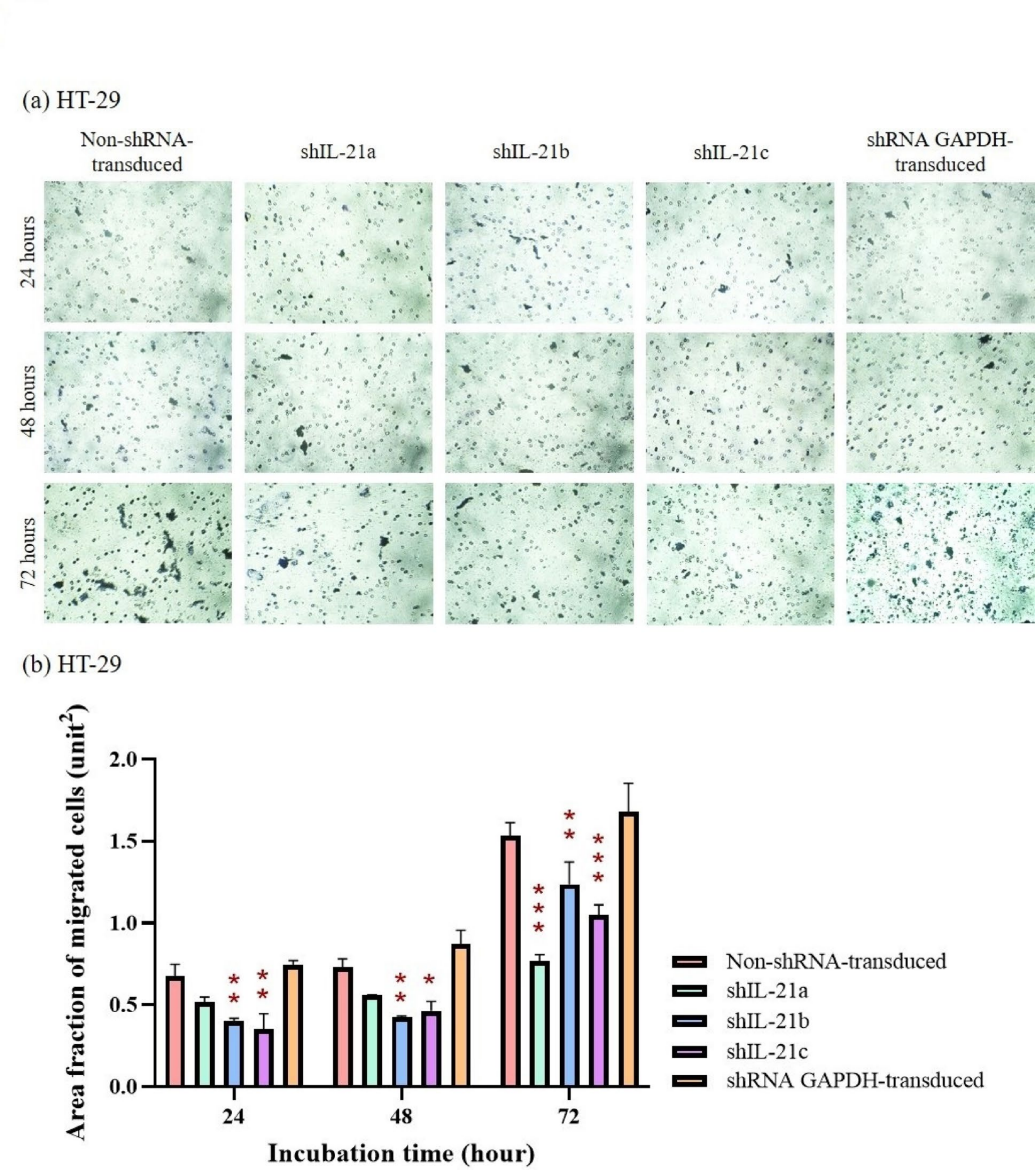
Migration analysis of shRNA-transduced IL-21-silenced HT29 cells. (**a**) Representative images of IL-21-silenced HT29 cells that migrated through an 8.0 µm pore size polyester membrane, captured using an Optika XDS-2 inverted microscope at 200 × magnification. (**b**) Area fractions of migrated IL-21-silenced HT29 cells stained using the Hemacolor^®^ Rapid Staining of Blood Smear Kit at 24, 48 and 72 h of incubation, quantified using ImageJ software. Data are presented as mean ± SD of three independent experiments. *P < 0.05, **P < 0.01, ***P < 0.0001

Similarly, shRNA-transduced IL-21-silenced HT-29 cells exhibited a lower area fraction of migrated cells than their respective controls at all time points (Fig. 12a, b). At 24 h, the area fraction of migrated IL-21-silenced HT-29 cells was 0.40 (*P* < 0.01; shIL-21b) and 0.35 unit^2^ (*P* < 0.01; shIL-21c). At 48 h, the migration levels slightly increased to 0.43 (*P* < 0.01; shIL-21b) and 0.46 (P < 0.05; shIL-21c) units. However, at 72 h, IL-21-silenced HT-29 cells still displayed significantly lower migrations, with area fractions of 0.77 (*P* < 0.001; shIL-21a), 1.24 (*P* < 0.01; shIL-21b), and 1.05 unit^2^ (*P* < 0.001; shIL-21c), whereas the control and shRNA GAPDH-transduced HT-29 cells achieved 1.53 and 1.68 unit^2^, respectively. In summary, the Transwell migration assay verified that shRNA-transduced IL-21 gene silencing significantly reduced the chemotactic capability of both HCT-116 and HT-29 cells, with a more pronounced effect in HCT-116 cells.

### Expression levels of proliferation and migration markers in shRNA-transduced IL-21-silenced HCT-116 and HT-29 cells

**Fig. 13:**
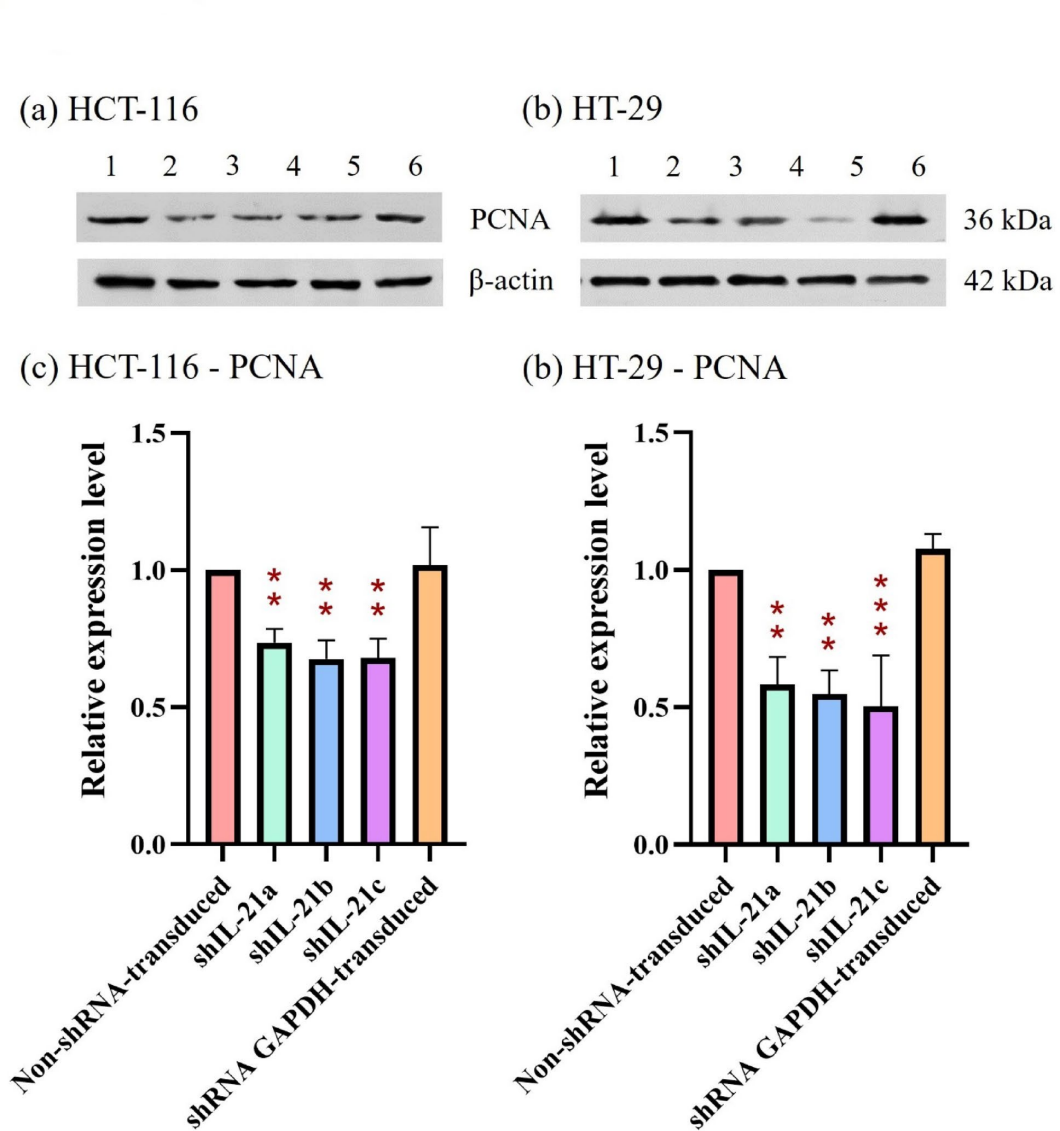
PCNA protein expression in shRNA-transduced IL-21-silenced HCT-116 and HT-29 cells. Western blot analysis of PCNA protein expression in IL-21-silenced (**a**) HCT-116 and (**b**) HT-29 cells after puromycin selection. Lane 1: non-shRNA-transduced; Lane 2: shIL-21a; Lane 3: shIL-21b; Lane 4: shIL-21c; Lane 5: shRNA GAPDH-transduced cells. Densitometric analysis of PCNA expression levels in IL-21-silenced (**c**) HCT-116 and (**d**) HT-29 cells was performed using ImageJ software. Data are presented as mean ± SD of three independent experiments and normalised to β-actin as a loading control. **P < 0.01, ***P < 0.001

**Fig. 14:**
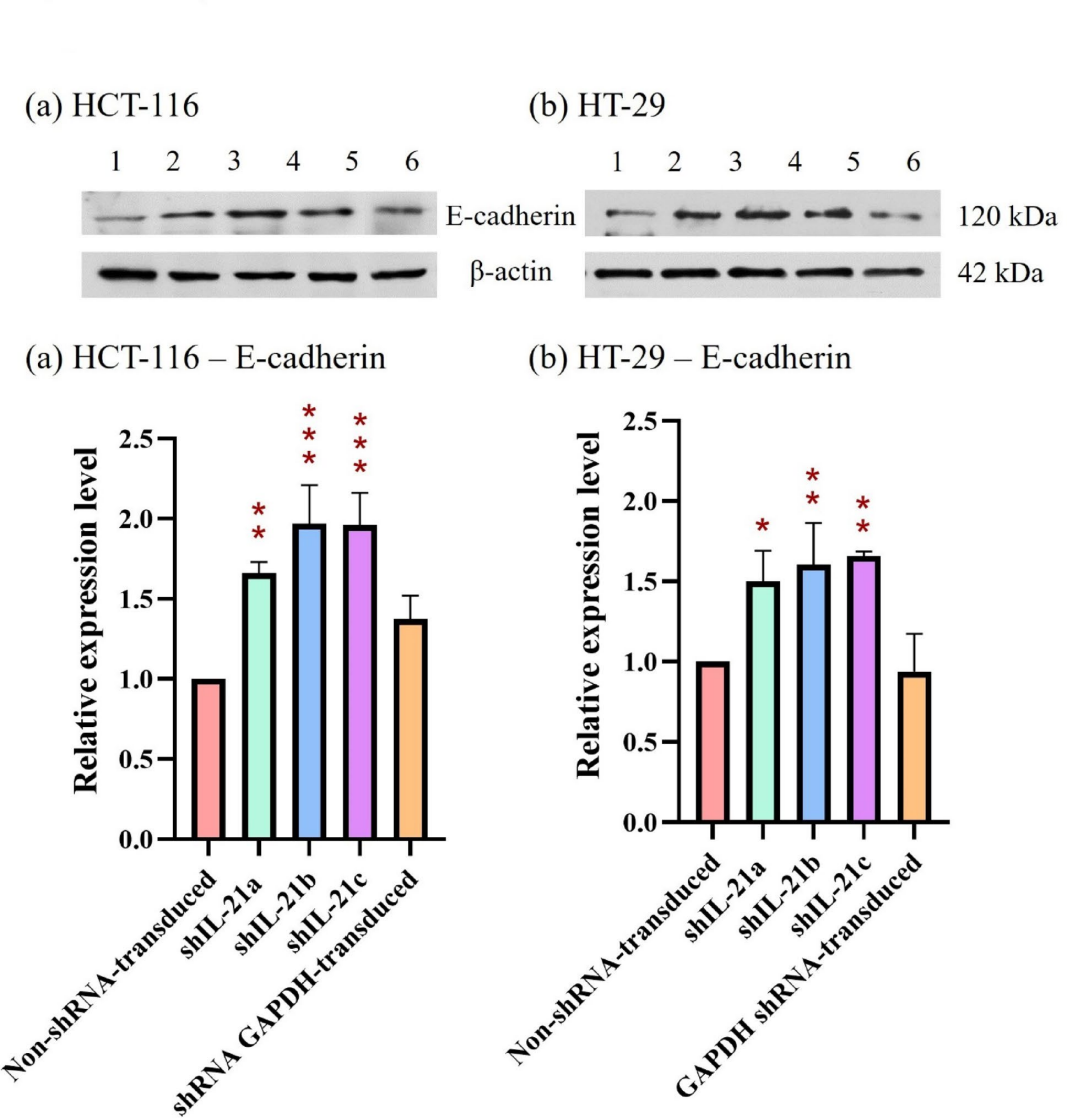
E-cadherin protein expression in shRNA-transduced IL-21-silenced HCT-116 and HT-29 cells. Western blot analysis of E-cadherin protein expression in IL-21-silenced (**a**) HCT-116 and (**b**) HT-29 cells after puromycin selection. Lane 1: non-shRNA-transduced; Lane 2: shIL-21a; Lane 3: shIL-21b; Lane 4: shIL-21c; Lane 5: shRNA GAPDH-transduced cells. Densitometric analysis of E-cadherin expression levels in IL-21-silenced (**c**) HCT-116 and (**d**) HT-29 cells was performed using ImageJ software. Data are presented as mean ± SD of three independent experiments and normalised to β-actin as a loading control. *P < 0.05, **P < 0.01, ***P < 0.001

**Fig. 15:**
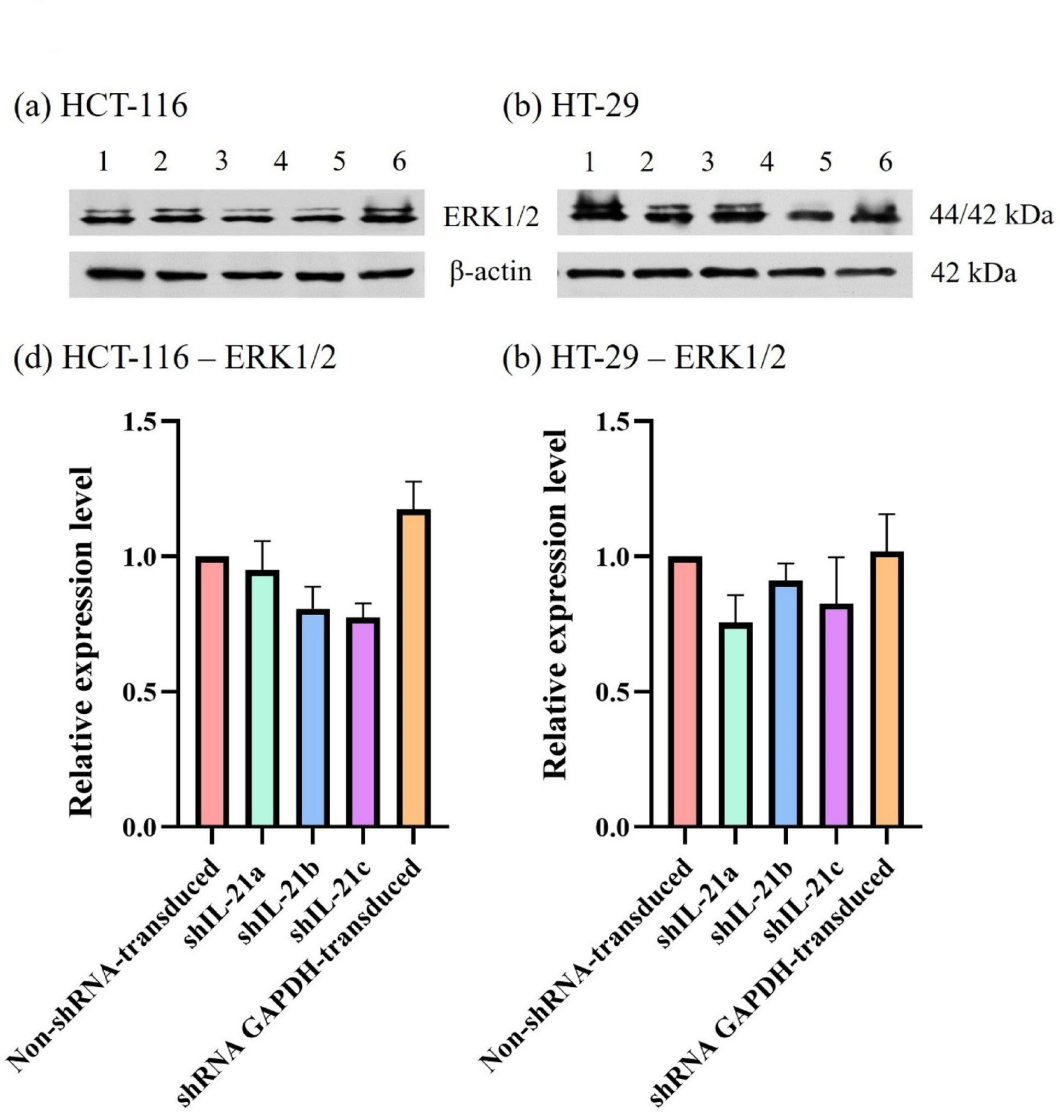
ERK1/2 protein expression in shRNA-transduced IL-21-silenced HCT-116 and HT-29 cells. Western blot analysis of ERK1/2 protein expression in IL-21-silenced (**a**) HCT-116 and (**b**) HT-29 cells after puromycin selection. Lane 1: non-shRNA-transduced; Lane 2: shIL-21a; Lane 3: shIL-21b; Lane 4: shIL-21c; Lane 5: shRNA GAPDH-transduced cells. Densitometric analysis of ERK1/2 expression levels in IL-21-silenced (**c**) HCT-116 and (**d**) HT-29 cells was performed using ImageJ software. Data are presented as mean ± SD of three independent experiments and normalised to β-actin as a loading control

**Fig. 16:**
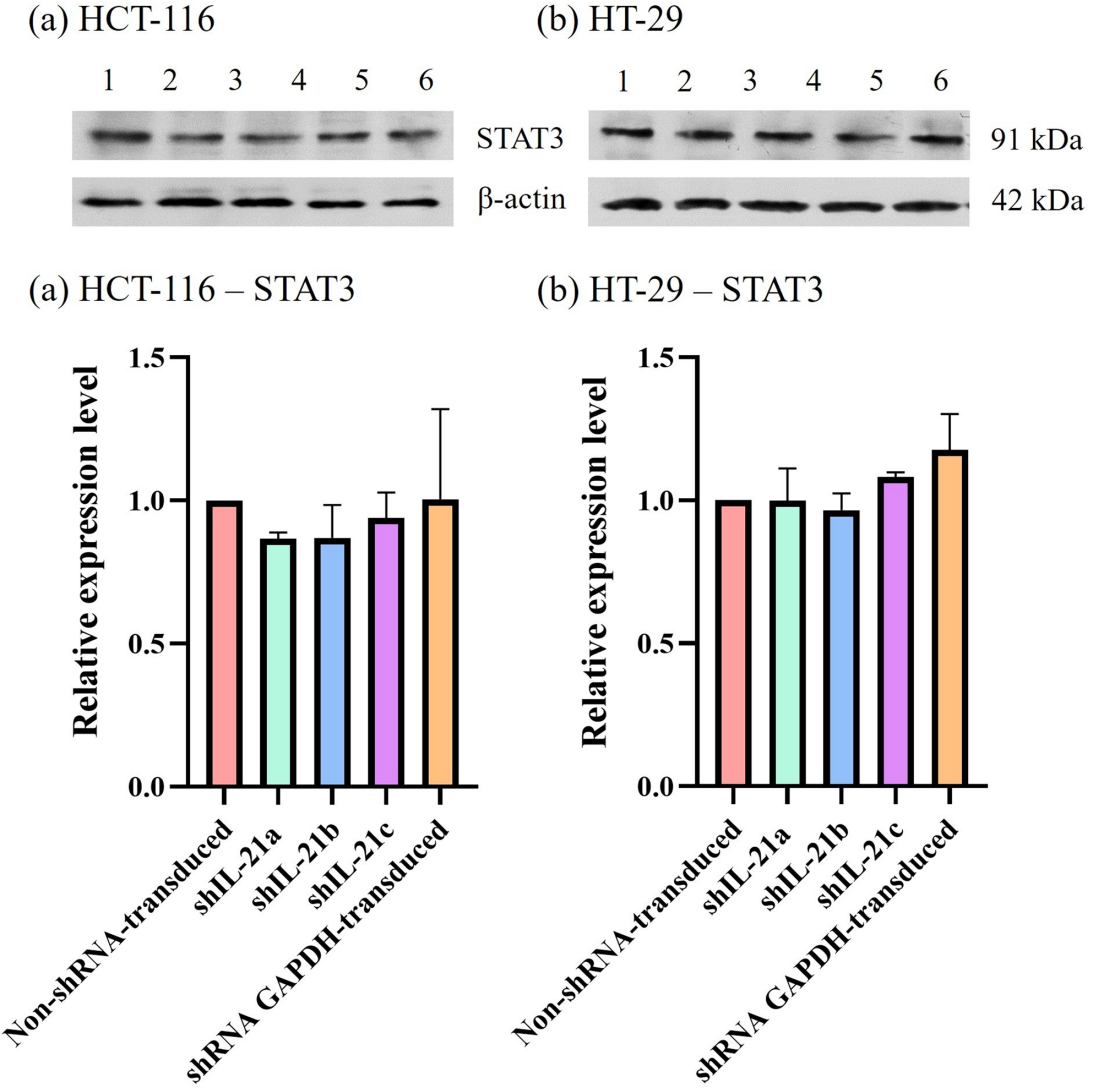
STAT3 protein expression in shRNA-transduced IL-21-silenced HCT-116 and HT-29 cells. Western blot analysis of STAT3 protein expression in IL-21-silenced (**a**) HCT-116 and (**b**) HT-29 cells after puromycin selection. Lane 1: non-shRNA-transduced; Lane 2: shIL-21a; Lane 3: shIL-21b; Lane 4: shIL-21c; Lane 5: shRNA GAPDH-transduced cells. Densitometric analysis of STAT3 expression levels in IL-21-silenced (**c**) HCT-116 and (**d**) HT-29 cells was performed using ImageJ software. Data are presented as mean ± SD of three independent experiments and normalised to β-actin as a loading control

**Fig. 17:**
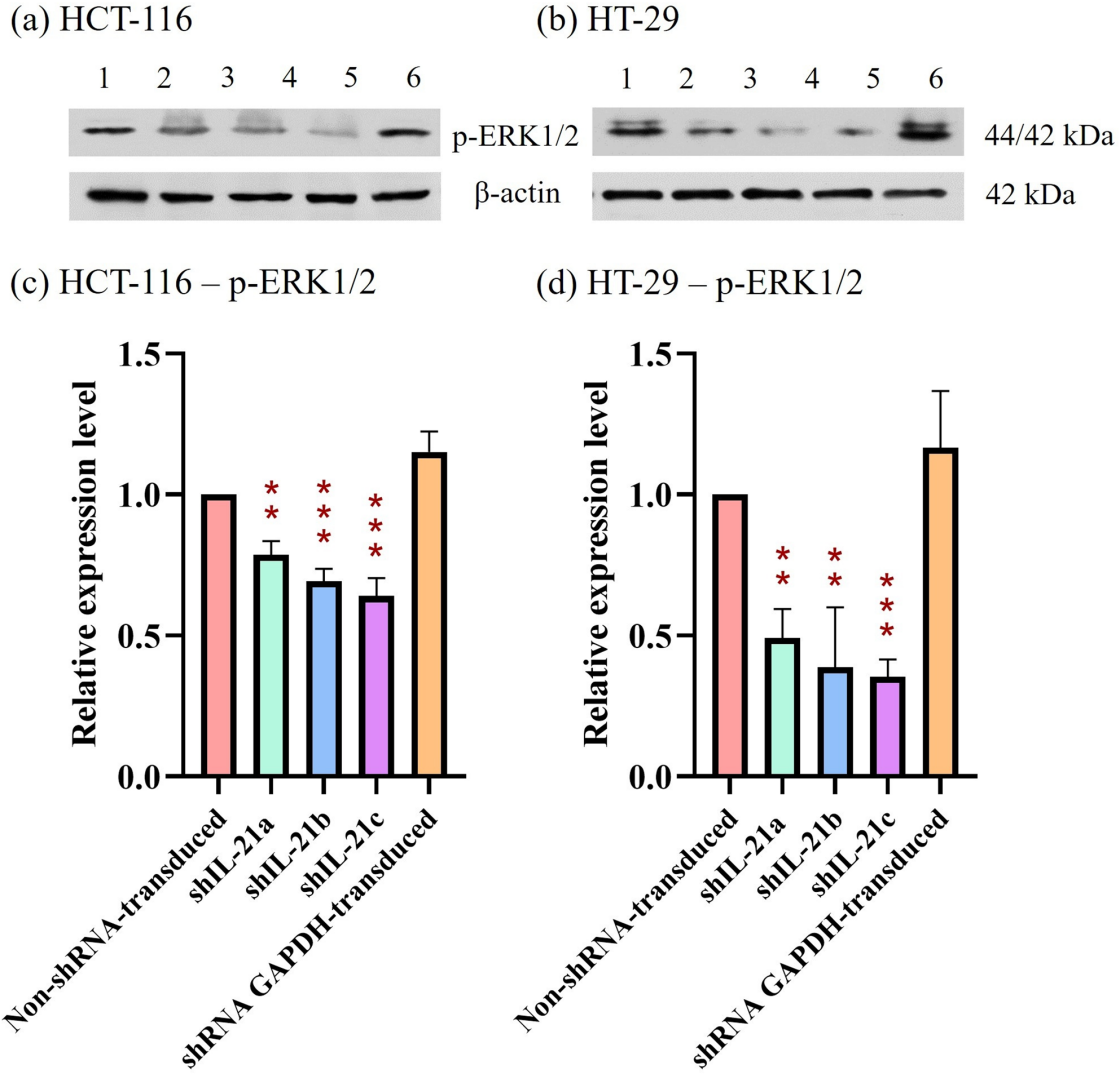
p-ERK1/2 protein expression in shRNA-transduced IL-21-silenced HCT-116 and HT-29 cells. Western blot analysis of p-ERK1/2 protein expression in IL-21-silenced (**a**) HCT-116 and (**b**) HT-29 cells after puromycin selection. Lane 1: non-shRNA-transduced; Lane 2: shIL-21a; Lane 3: shIL-21b; Lane 4: shIL-21c; Lane 5: shRNA GAPDH-transduced cells. Densitometric analysis of p-ERK1/2 expression levels in IL-21-silenced (**c**) HCT-116 and (**d**) HT-29 cells was performed using ImageJ software. Data are presented as mean ± SD of three independent experiments and normalised to β-actin as a loading control. **P < 0.01, ***P < 0.001

**Fig. 18:**
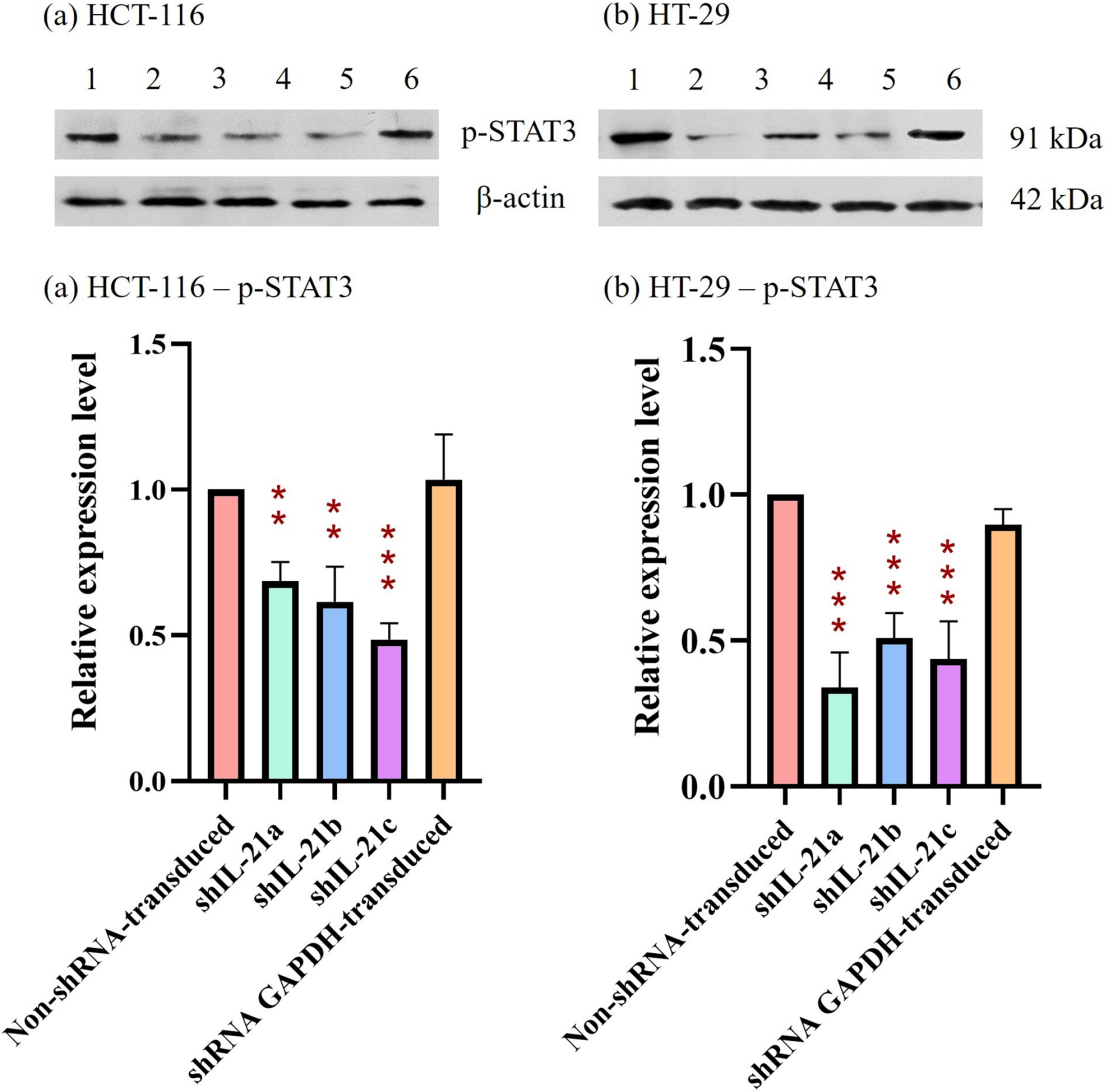
p-STAT3 protein expression in shRNA-transduced IL-21-silenced HCT-116 and HT-29 cells. Western blot analysis of p-STAT3 protein expression in IL-21-silenced (**a**) HCT-116 and (**b**) HT-29 cells after puromycin selection. Lane 1: non-shRNA-transduced; Lane 2: shIL-21a; Lane 3: shIL-21b; Lane 4: shIL-21c; Lane 5: shRNA GAPDH-transduced cells. Densitometric analysis of p-STAT3 expression levels in IL-21-silenced (**c**) HCT-116 and (**d**) HT-29 cells was performed using ImageJ software. Data are presented as mean ± SD of three independent experiments and normalised to β-actin as a loading control. ***P < 0.001

The effect of shRNA-transduced IL-21 gene silencing on the expression of the proliferation marker, proliferating cell nuclear antigen (PCNA), revealed a downregulation in IL-21-silenced HCT-116 cells to 0.74-fold (*P* < 0.01; shIL-21a) and 0.68-fold (*P* < 0.01; shIL-21b and shIL-21c) (Fig. 13). Similarly, IL-21-silenced HT-29 cells exhibited a decrease in PCNA expression to 0.58-fold (*P* < 0.01; shIL-21a), 0.55-fold (*P* < 0.01; shIL-21b), and 0.50-fold (*P* < 0.001; shIL-21c) relative to their respective non-shRNA-transduced cells. In contrast, E-cadherin expression was significantly upregulated in both IL-21-silenced HCT-116 and HT-29 cells (Fig. 14). IL-21-silenced HCT-116 cells displayed an increase of 1.66-fold (*P* < 0.01; shIL-21a), 1.97-fold (*P* < 0.001; shIL-21b), and 1.96-fold (*P* < 0.001; shIL-21c), whereas IL-21-silenced HT-29 cells showed an increase of 1.50-fold (*P* < 0.05; shIL-21a), 1.61-fold (*P* < 0.01; shIL-21b), and 1.66-fold (*P* < 0.01; shIL-21c) relative to their respective controls. The expression levels of ERK1/2 (Fig. 15) and STAT3 (Fig. 16) were not significantly altered in both IL-21-silenced HCT-116 and HT-29 cells. However, a significant downregulation of phosphorylated ERK1/2 (p-ERK1/2) expression was observed in IL-21-silenced HCT-116, with expression levels reduced to 0.79-fold (*P* < 0.01; shIL-21a), 0.69-fold (*P* < 0.001; shIL-21b), and 0.64-fold (*P* < 0.001; shIL-21c) relative to the control (Fig. 17). Similarly, IL-21-silenced HT-29 cells showed decreased p-ERK1/2 expression of 0.49-fold (*P* < 0.01; shIL-21a), 0.39-fold (*P* < 0.01; shIL-21b), and 0.35-fold (*P* < 0.001; shIL-21c) relative to the control. The phosphorylated STAT3 (p-STAT3) expression was also significantly downregulated in IL-21-silenced HCT-116 cells to 0.69-fold (*P* < 0.01; shIL-21a), 0.62-fold (*P* < 0.01; shIL-21b), and 0.49-fold (*P* < 0.001; shIL-21c). IL-21-silenced HT-29 cells displayed similar reductions, with p-STAT3 expression decreasing to 0.34-fold (*P* < 0.001; shIL-21a), 0.51-fold (*P* < 0.001; shIL-21b), and 0.44-fold (*P* < 0.001; shIL-21c) relative to the control (Fig. 18). Overall, these results indicate that shRNA-transduced IL-21 gene silencing led to upregulation of E-cadherin and reduced PCNA expression in both HCT-116 and HT-29 cells. Furthermore, IL-21 gene silencing suppressed ERK1/2 and STAT3 activation, as indicated by reduced phosphorylation (p-ERK1/2 and p-STAT3, respectively), rather than reducing their total protein expression.

## Discussion

**Fig. 19:**
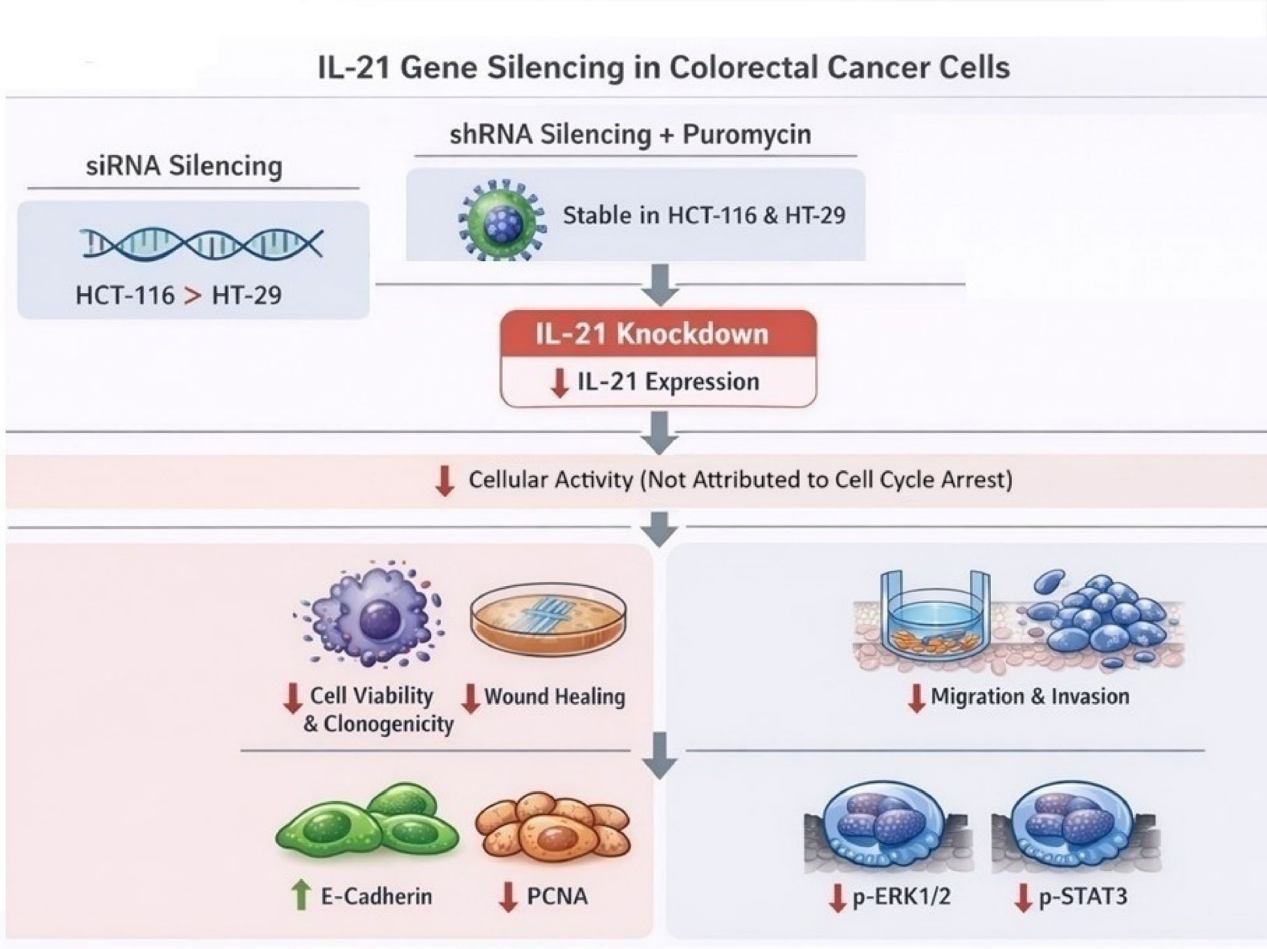
The experimental workflow and the overall findings of this study.

Overall, siRNA-mediated IL-21 gene silencing was more effective in reducing IL-21 protein expression in HCT-116 than in HT-29 cells. In contrast, shRNA-transduced IL-21 gene silencing, under puromycin selection, achieved efficient and stable gene silencing in both cell lines. The reduced viability and clonogenicity observed in shRNA-transduced IL-21-silenced cells confirmed the effectiveness of this approach. The reduction in cell viability and proliferation rate was not attributed to cell cycle arrest but rather to reduced cellular activity, as evidenced by the diminished wound-healing ability of IL-21-silenced HCT-116 cells. Additionally, the Transwell migration assay demonstrated that IL-21 gene silencing impaired the chemotactic capability of both HCT-116 and HT-29 cells. Notably, IL-21 gene silencing led to upregulation of E-cadherin and decreased PCNA protein expression in both cell lines, suggesting impaired cell activity due to reduced receptiveness of epithelial-to-mesenchymal transition (EMT) initiation. IL-21 gene silencing also inhibited ERK1/2 and STAT3 phosphorylation rather than reducing overall ERK1/2 and STAT3 protein levels. The experimental workflow and the overall findings of this study are summarised in Fig. 19.

The findings from IL-21 gene silencing in HCT-1116 and HT-29 cells using siRNA and shRNA approaches revealed that HCT-116 cells were more responsive to siRNA-mediated gene silencing, whereas shRNA-transduced gene silencing was equally effective in both cell lines. This difference may be attributed to intrinsic and extrinsic factors. Intrinsic factors include cell doubling time and morphology. A previous study suggested that variations in cell doubling time can influence the dilution effect of siRNAs due to cell division [18]. HCT-116 cells, with a shorter doubling time (~ 18 h) compared to HT-29 cells (~ 24 h), exhibited a stronger genesilencing effect when using siRNA. Additionally, cell morphology may affect siRNA transfection efficiency. A previous study showed that well-spread, elongated cells facilitate gene transfection by enhancing the uptake of cationic complexes [19]. The HCT-116 cells, characterised by a neurite-like morphology with an extensive and elongated surface area, provided a larger exposed surface area for siRNA uptake, resulting in higher transfection and gene silencing efficiency compared to the round-shaped HT-29 cells. The increased siRNA transfection efficiency and gene silencing observed in HCT-116 cells can also be attributed to the stability and abundance of the target gene mRNA.

Extrinsic factors influencing gene silencing efficiency include cell confluency, the logarithmic growth phase, the optimal amount of transfection reagent and siRNA complexes to balance high gene silencing efficiency with low toxicity, and the presence of serum during transfection, which can interfere with siRNA complex formation with cationic lipid-based transfection reagents. Unlike siRNA, the pGIPZ lentiviral vector used for shRNA-mediated IL-21 gene silencing contains two key markers: TurboGFP and the puromycin-resistance gene, which facilitate selection. TurboGFP expression enables visual identification of shRNA-expressing cells, while the puromycin-resistance gene ensures the survival and propagation of stably transduced cells under antibiotic selection. Therefore, we used the percentage of GFP-positive cells to determine the transduction efficiency of the three different shRNA constructs and controls. Both cell lines displayed similar results, with HCT-116 exhibiting slightly higher shRNA transduction efficiency than HT-29. This difference may be attributed to variation in the expression of cellular receptors between the two cell lines, leading to different cell tropisms in response to lentiviral transduction [20].

shRNA molecules are constitutively transcribed in the nucleus before undergoing further processing, potentially reducing off-target effects compared to siRNA. In contrast, exogenously introduced siRNA molecules may be degraded in the cytoplasm before binding to target mRNA [21]. Additionally, viral vectors integrate shRNA plasmids into the host genome, enabling stable, long-term expression and consistent gene silencing. In contrast, transient siRNA duplexes provide only temporary IL-21 gene silencing, making shRNA-transduced gene silencing more efficient. Another key factor influencing the efficiency of gene silencing is the target mRNA turnover rate [18, 22]. A previous study suggested that genes with a lower turnover rate tend to have a longer half-life, making them more susceptible to siRNA-mediated gene silencing owing to prolonged target availability [22]. Conversely, genes with high mRNA turnover are less targetable and more resistant to siRNA repression [23]. Cytokines, such as IL-21, have short half-lives, making them less susceptible to siRNA-mediated gene silencing [24]. However, shRNA transduction, which ensures constitutive shRNA expression, overcomes this issue by prolonging gene silencing, thereby enhancing mRNA targetability.

The use of shRNA may pose toxicity concerns owing to potential overexpression. In contrast, siRNA allows for more precise dosage control, and its construct sequence can be more easily modified to enhance target specificity, whereas chemical modifications for shRNA remain relatively limited [25]. Therefore, evaluating the relative advantages and disadvantages of synthetic siRNAs versus endogenously expressed shRNAs is crucial before advancing RNAi-based therapies into clinical applications for cancer treatment [26]. Given the need for consistent and reproducible loss-of-function genesilencing approaches, we employed both siRNA and shRNA to achieve stable silencing of IL-21 gene, enabling downstream analyses of cell proliferation, viability, and clonogenicity. In addition to RNAi strategies, the downregulation of IL-21 and its receptor (IL-21R) in cancerous environments, particularly in solid tumours, has also been associated with activation of the Wnt/β-catenin signalling pathway [27]. Activation of this pathway in Non-small cell lung cancer (NSCLC) leads to decreased expression of IL-21 and IL-21R, whereas inhibition of Wnt/β-catenin signalling can restore their expression levels. Furthermore, increased programmed death-ligand 1 (PD-L1) expression has been reported to negatively correlate with IL-21/IL-21R levels in NSCLC, suggesting that signalling pathways promoting immune checkpoint evasion may also contribute to IL-21 suppression. Investigation of these mechanisms, including the use of Wnt/β-catenin inhibitors, will be explored in future studies.

Cancer cell proliferation refers to an increase in cell number caused by uncontrolled growth and division [28]. Targeting proliferative signals remains a key objective in cancer treatment development. The cell cycle, which refers to a sequence of phases in cell division, consists of distinct stages: gap 1 (G1), synthesis (S), gap 2 (G2), and mitotic (M) phases, all of which are vital for the viability and development of eukaryotic cells [29]. Cell cycle analysis was used to assess the distribution of the cell population across these phases and determine the impact of IL-21 gene silencing on cell cycle progression. The cell cycle is regulated by a cascade of CDK activation and checkpoint controls [30]. Notably, no cell cycle arrest was observed in either HCT-116 or HT-29 cells upon IL-21 gene silencing, despite reductions in cell viability and clonogenicity. This suggests that the decrease in proliferative activity in IL-21-silenced cells is not driven by direct alterations in cell-cycle regulation but rather by disruptions in cellular metabolic processes. The rapid growth of cancer cells is highly dependent on the upregulation of growth signals to meet their increased energy and biosynthetic demands. Disrupting key cellular metabolic pathways, potentially through IL-21 gene silencing, can lead to reduced proliferation or even apoptosis if sustained over time [31]. One such metabolic adaptation in cancer cells is the Warburg effect, which is characterised by enhanced glucose uptake and utilisation [32]. Although no direct evidence currently links IL-21 to the Warburg effect in cancer cells, several studies have shown that proinflammatory cytokines can induce glucose metabolism in human cells. Based on existing literature, it is plausible that IL-21 signalling may regulate the proliferative activity of colorectal cancer cells by influencing metabolic pathways. However, further studies are needed to confirm this hypothesis.

In addition to functional assays, we assessed the impact of IL-21 gene silencing on proliferative characteristics of HCT-116 and HT-29 cells by evaluating PCNA protein expression. PCNA is a nuclear non-histone protein essential for DNA synthesis. It serves as an accessory protein for DNA polymerase α with peak expression during the G1/S phase of the cell cycle. PCNA is often used as a proliferation marker in diagnostic and prognostic studies, as its expression accurately reflects the rate of cell proliferation and DNA synthesis in many solid tumours, including colorectal cancer [33, 34]. Western blot analysis revealed downregulation of PCNA expression in both IL-21-silenced HCT-116 and HT-29 cell lines. This finding suggests that IL-21 gene silencing suppresses the proliferative characteristics of colorectal cancer cells. Although no cell cycle arrest was observed, these results suggest that other cancer hallmarks, such as invasion, activation and metastasis, may be affected by IL-21 gene silencing.

The ability to activate cell migration is a hallmark of cancer and is of significant research interest owing to its role in metastasis, the leading cause of mortality among cancer patients [35]. Cell migration is a fundamental cellular process essential for development and homeostasis [36]. In cancer pathogenesis, migration is crucial for tumour growth and dissemination, serving as a prerequisite for invasion and metastasis. In vitro assays can quantify cancer cell migration, providing insights into the metastatic potential of tumours [37]. There are two primary modes of cancer cell migration: individual and collective. Individual cell migration occurs through cytoskeletal activity without interaction with neighbouring cells, whereas collective cell migration involves coordinated cytoskeletal movement with neighbouring cells while maintaining cell–cell junctions. Individual cell migration is particularly implicated in the early stages of cancer metastasis. The Transwell migration assay assesses this ability by measuring the movement of individual cells toward a chemoattractant [38]. In our study, a greater number of IL-21-silenced HCT-116 cells migrated through the porous membrane of Transwell inserts compared to IL-21-silenced HT-29 cells, likely reflecting the greater aggressiveness of HCT-116 cells. Nevertheless, both IL-21-silenced HCT-116 and HT-29 cells exhibited reduced migratory capacity compared to control cells, indicating that the IL-21 gene impairs chemotactic ability in both cell types. In contrast, collective cell migration facilitates the coordinated movement of groups—often involving immobile cell types—to maintain proper cell distribution and tissue organisation [37]. The wound healing assay, commonly used to study collective migration in cell monolayers [38, 39], demonstrated that IL-21 gene silencing reduced the wound healing rate in both HCT-116 and HT-29 cells. These findings suggest that IL-21 supports collective migration in colorectal cancer cells.

E-cadherin, a key cell adhesion molecule, is abundant in epithelial adherens junctions, where it facilitates epithelial sheet formation and maintains cell quiescence. In cancers, E-cadherin functions as a tumour suppressor, reinforcing intercellular junctions and suppressing EMT during tumour progression [35]. Conversely, the loss of E-cadherin is associated with enhanced cancer cell migration and invasiveness [40]. Western blot analysis revealed upregulation of E-cadherin and downregulation of PCNA in IL-21-silenced HCT-116 and HT-29 cells, suggesting that cell adhesion was restored while proliferation was suppressed. The restoration of the E-cadherin adhesion complex in IL-21-silenced HCT-116 and HT-29 cells inhibited their transition to a migratory phenotype, preserving epithelial morphology and maintaining cell–cell interactions, thereby suppressing proliferation [41]. Consequently, the upregulation of E-cadherin and downregulation of PCNA protein expression in IL-21-silenced cells suggest a lower propensity for EMT initiation.

IL-21 is known to signal through a heterodimeric receptor complex consisting of IL-21R and the common γ chain/IL-2Rγ [42]. Similar to other γ chain cytokines, IL-21 transduces molecular signals through three major pathways: ERK/MAPK, AKT/PI3K, and JAK/STAT [43]. Western blot analysis revealed that IL-21 gene silencing suppressed phosphorylation of ERK1/2 and STAT3 in HCT-116 and HT-29 cells. ERK1/2, a serine-threonine kinase, plays a crucial role in signalling cascades—transmitting extracellular signals to intracellular targets. Hyperactivation of the ERK/MAPK signalling pathway contributes to tumour proliferation, invasion, metastasis, extracellular matrix degradation, and angiogenesis [44]. IL-21 induces ERK1/2 activation, thereby promoting cell proliferation [45]. Additionally, ERK1/2 activation is implicated in IL-21-induced cytokine production in leukaemia cells and monocytes [46]. In contrast, STAT3 activation is more frequent than STAT5 activation in solid tumours, although both contribute to cancer malignancy [47]. STAT3 is widely recognised as an oncogene owing to its constitutive activation in multiple malignant solid tumours, including breast, prostate, renal cell, brain, and ovarian cancers [48]. The downstream targets of STAT3 include genes involved in tumour development, such as proliferation, invasion, angiogenesis, and elevated cytokine expression [43, 49]. Additionally, activated STAT3 has been identified as a potential biomarker in patients with colorectal cancer and is known to be activated by IL-21, a STAT3 target gene [48]. This creates an autocrine positive feedback loop for IL-21-mediated signalling. A previous study demonstrated that IL-21 promoted cell growth in ALK-positive anaplastic large-cell lymphoma by activating JAK3/STAT3 [50]. Consistent with our findings, IL-21-stimulated proliferation via STAT3 has been observed in rheumatoid arthritis fibroblast-like synoviocytes [51]. These findings support the hypothesis that IL-21 enhances cell proliferation and migration via ERK1/2- and STAT3-dependent pathways, and that this effect can be downregulated by IL-21 gene silencing.

## Conclusion

In summary, IL-21 regulates the proliferation, growth, and migration of HCT-116 and HT-29 cells via ERK1/2- and STAT3-dependent pathways by suppressing ERK1/2 and STAT3 activation, thereby providing insights into potential targets for future therapeutic strategies.

## Data Availability

Data are available upon reasonable request from the authors.
